# Alpha-linolenic acid stabilizes HIF-1 α and downregulates FASN to promote mitochondrial apoptosis for mammary gland chemoprevention

**DOI:** 10.18632/oncotarget.19551

**Published:** 2017-07-25

**Authors:** Subhadeep Roy, Atul Kumar Rawat, Shreesh Raj Sammi, Uma Devi, Manjari Singh, Swetlana Gautam, Rajnish Kumar Yadav, Jitendra Kumar Rawat, Lakhveer Singh, Mohd. Nazam Ansari, Abdulaziz S. Saeedan, Rakesh Pandey, Dinesh Kumar, Gaurav Kaithwas

**Affiliations:** ^1^ Department of Pharmaceutical Sciences, Babasaheb Bhimrao Ambedkar University, Lucknow (UP), India; ^2^ Central for Biomedical Research, Sanjay Gandhi Post Graduate Institute of Medical Sciences Campus, Lucknow (UP), India; ^3^ Department of Microbial Technology and Nematology, CSIR-Central Institute of Medicinal and Aromatic Plants, Lucknow (UP), India; ^4^ Department of Pharmaceutical Sciences, Faculty of Health and Medical Sciences, Sam Higginbottom Institute of Agricultural Sciences and Technology, Allahabad (UP), India; ^5^ Department of Pharmacology, College of Pharmacy, Prince Sattam Bin Abdulaziz University, Al-Kharj, KSA

**Keywords:** alpha linolenic acid, apoptosis, polyunsaturated fatty acid, hypoxia, fatty acid synthase

## Abstract

Alpha linolenic acid is an essential polyunsaturated fatty acid and is reported to have the anti-cancer potential with no defined hypothesis or mechanism/s. Henceforth present study was in-quested to validate the effect of alpha linolenic acid on mitochondrial apoptosis, hypoxic microenvironment and de novo fatty acid synthesis using *in-vitro* and *in-vivo* studies. The IC_50_ value of alpha linolenic acid was recorded to be 17.55μM against ER+MCF-7 cells. Treatment with alpha linolenic acid was evident for the presence of early and late apoptotic signals along with mitochondrial depolarization, when studied through acridine orange/ethidium bromide and JC-1 staining. Alpha linolenic acid arrested the cell cycle in G2/M phase. Subsequently, the *in-vivo* efficacy was examined against 7, 12-dimethylbenz anthracene induced carcinogenesis. Treatment with alpha linolenic acid demarcated significant effect upon the cellular proliferation as evidenced through decreased in alveolar bud count, restoration of the histopathological architecture and loss of tumor micro vessels. Alpha linolenic acid restored the metabolic changes to normal when scrutinized through ^1^H NMR studies. The immunoblotting and qRT-PCR studies revealed participation of mitochondrial mediated death apoptosis pathway and curtailment of hypoxic microenvironment after treatment with alpha linolenic acid. With all above, it was concluded that alpha linolenic acid mediates mitochondrial apoptosis, curtails hypoxic microenvironment along with inhibition of de novo fatty acid synthesis to impart anticancer effects.

## INTRODUCTION

α-Linolenic acid (ALA) (18: 3, ω-3) is an essential polyunsaturated fatty acid (PUFA). ALA (18: 3, ω-3) cannot be synthesized by the human body and needs to be obtained from dietary sources [1]. ALA is the major plant-based PUFA and is found in walnuts, flaxseeds, hemp seeds and their oils. ALA is also found in rapeseed (canola) oil; and to smaller amounts in soya oil and green-leafy vegetables [2]. ALA is metabolized by series of desaturation and elongation reactions of long chain fatty acids, among which eicosapentanoic acid (EPA, 20:5, ω-3) and docosahexaenoic acid (DHA, 22:6 ω-3) are of prime biological importance [3]. EPA and DHA are vital in regulating membrane fluidity, protein and cellular functions, eicosanoid metabolism, gene expression and cell signaling [4]. EPA and DHA are obtained from fish oil or derived from plant lipids rich in ALA. EPA and DHA integrate a cascade that runs alongside and emulates with the inflammatory cascade governed by the arachidonic acid (AA) (20: 4, ω-6) metabolism [5]. A previous report has taken account that the EPA cascade softens the inflammatory effects of AA cascade, suggesting it as an anti-inflammatory agent [6].

Report have also elaborated that ω-3 fatty acids reduce prostate tumor growth, curtail histopathological progression and increase survival [7]. One of the previous study has also indicated that ALA actively suppresses the overexpression of HER2+ in mammary carcinomas [8]. It was also reported that overiectomized mice produce a significant reduction in tumor growth, when compared with a diet containing no flaxseed oil (rich source of ALA)[9]. Moreover, PUFAs are the integral component of cell membranes and are susceptible to peroxidation and degeneration causing genetic mutations, a critical mechanism for tumor growth [10].Therefore, ALA seems to be a promising chemotherapeutic agent with desirable characteristics. Although studies have reported anti-carcinogenic potential of ALA and ALA being a PUFA can moderate the cell membrane integrity in multiple ways, the mechanistic pathway behind the same is unanswered. Considering the same, the present study was proposed to explore the effects of ALA on mammary gland carcinoma with concomitant efforts to elucidate the possible mechanism beneath the same.

## RESULTS

### MTT

**Figure 1:**
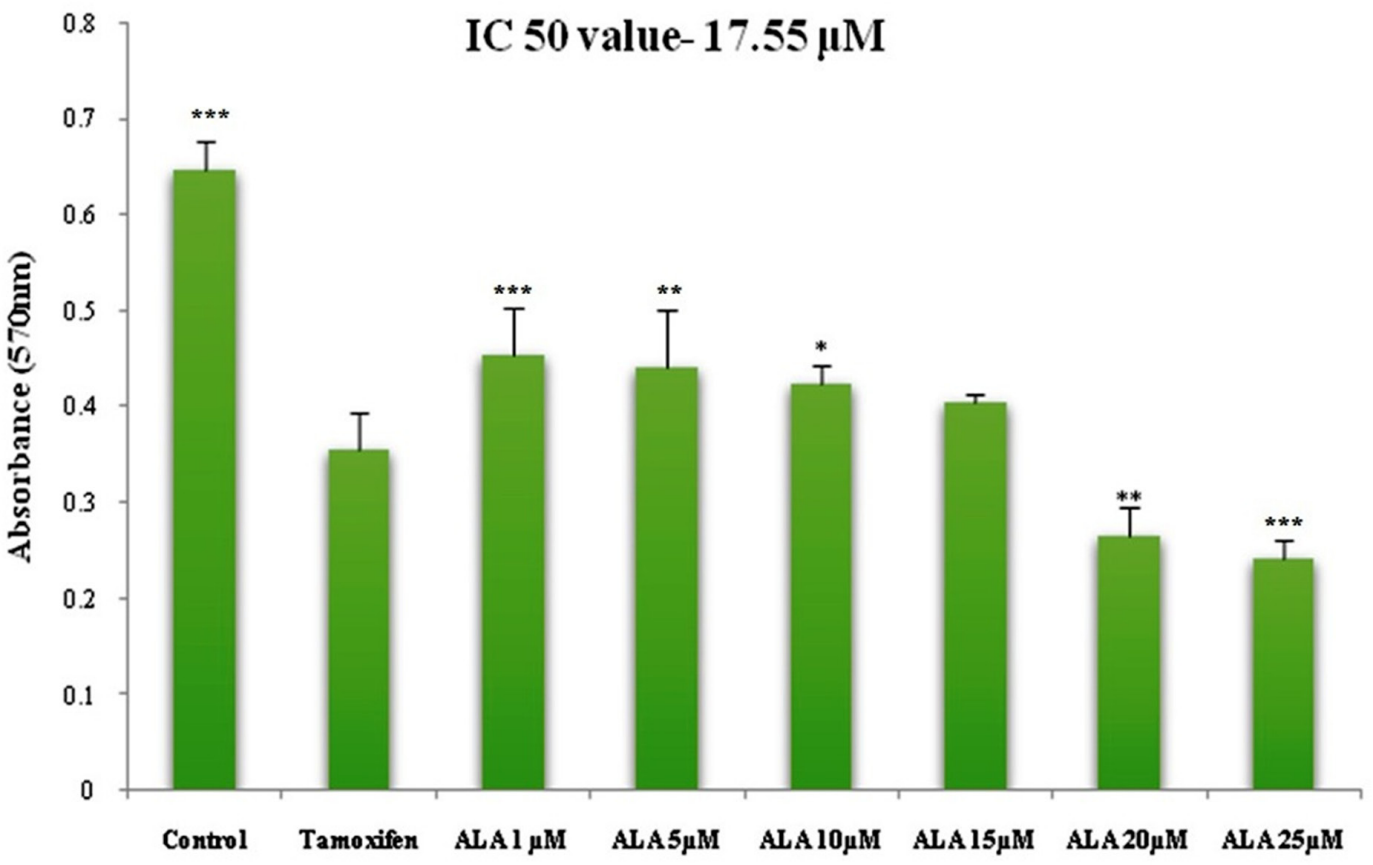
Cytotoxicity assessment of ALA Histogram reveals the effect of ALA (1μM, 5μM, 10μM, 15μM, 20μM and 25μM) on cell cytotoxicity by MTT assay on ER+MCF-7 cell line after 24 h incubation. The O.D. at 570 nm was compared with the untreated cell and TMX (27μM) treated cells. Reduction in the O.D. at 570 nm was observed in a dose dependent manner. Values are presented as mean ± SD and denote significant decreased in O.D. at 570 nm from control values. The comparisons are made by one-way ANOVA followed by Bonferroni multiple test.

ALA (1μM, 5μM, 10μM, 15μM, 20μM, 25μM) significantly inhibited the growth of ER+MCF-7 cells in the dose dependent manner. The IC_50_ of ALA was calculated to be 17.55 μM against ER+MCF-7 cells (Figure 1).

### Morphological studies for detection of apoptosis

**Figure 2:**
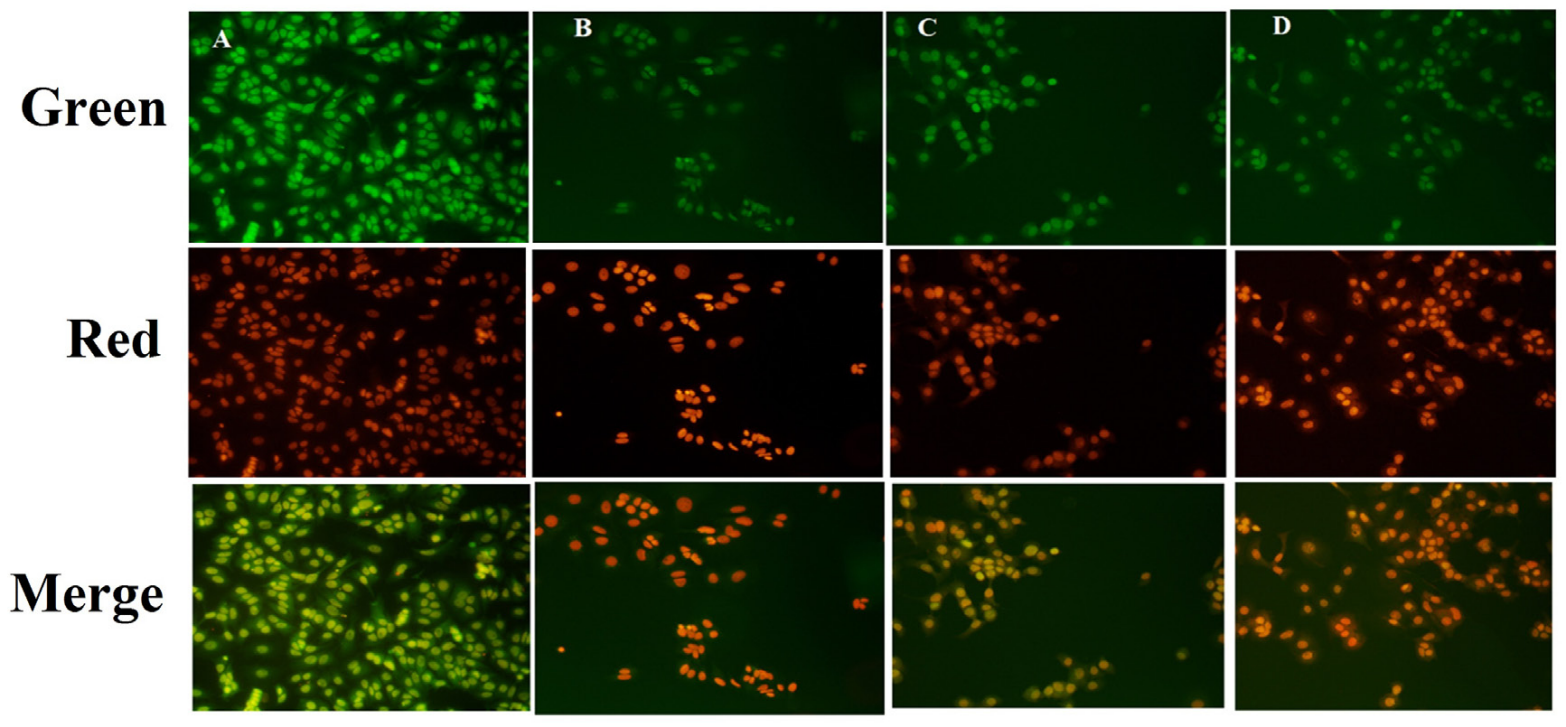
Effect of ALA on early apoptotic changes studied through AO/EtBr staining In fluorescence microscopic study of control **(A)**; standard: TMX **(B)**; ALA (1/2 IC_50_) **(C)**; ALA (IC_50_) **(D)** with dual staining [AO (100μg/ml): EtBr (100μg/ml) in 1:1 ratio]. The fluorescence was measured in three respective channels (green, red and merged) with 20X magnification, which reveals the morphological changes of apoptosis in experimental procedure after 18h drug treatment.

Fluorescence microscopic observations of the ER+MCF-7cells stained with acridine orange/ethidium bromide (AO/EtBr) (color-red or orange), revealed the presence of early and late apoptotic signals, including chromatin condensation, apoptotic body formation, membrane blebbing and fragmented nuclei. AO/EtBr staining affirmed late and early apoptotic changes in the ER+ MCF-7 cells induced by ALA treatment (Figure 2).

### Measurement of mitochondrial membrane potential

**Figure 3:**
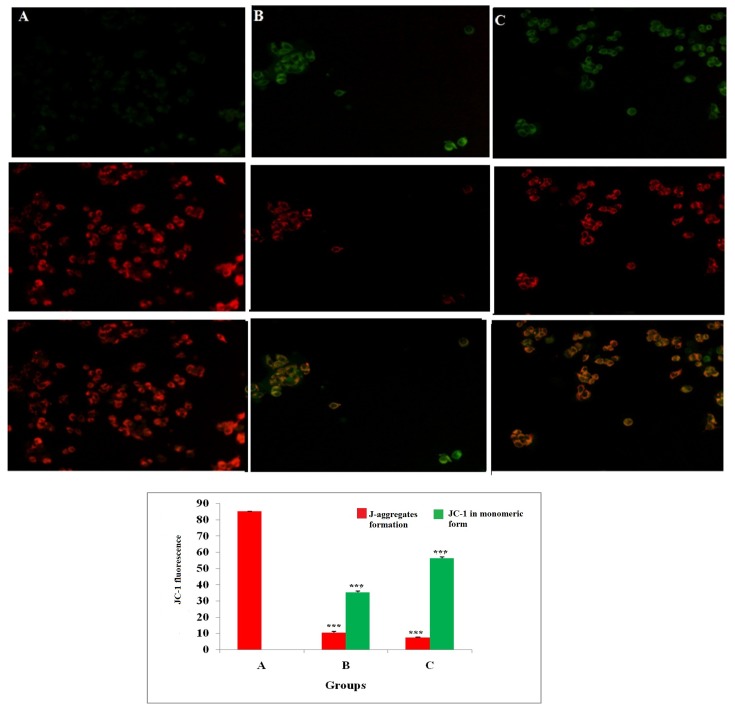
Effect of ALA on mitochondrial membrane potential (Δψ) ER+MCF-7 cells treated with control **(A)**; ALA (1/2 IC_50_) **(B)**; ALA (IC_50_) **(C)** for 18h and proceeded with JC-1 dye (5 mg/ml) for 1h. The cells were examined under inverted fluorescence microscope in three respective channels (green, red and merged). Internal accumulations of JC-1 aggregate in apoptotic cells are represented through change in ratio of orange to green fluorescence intensity in merged channel. Values are presented as mean ± SD and the comparisons are made on the basis of one-way ANOVA followed by Bonferroni multiple test.

JC-1 is a cationic dye used to study mitochondrial depolarization. Mitochondrial depolarization is indicated by increase in green fluorescence intensity and decrease in orange intensity due to failure in intracellular accumulation of J-aggregates and their monomeric form. This represents disruption of active mitochondrial membrane and loss of conformation in mitochondria permeability transition pore (MPTP). Treatment with ALA increased the uptake of JC-1 in cells, which was visualized through increase in green fluorescence intensity suggesting early apoptotic mitochondrial depolarization (Δψ) (Figure 3).

### Cell cycle analysis using propidium iodide (PI)

**Figure 4:**
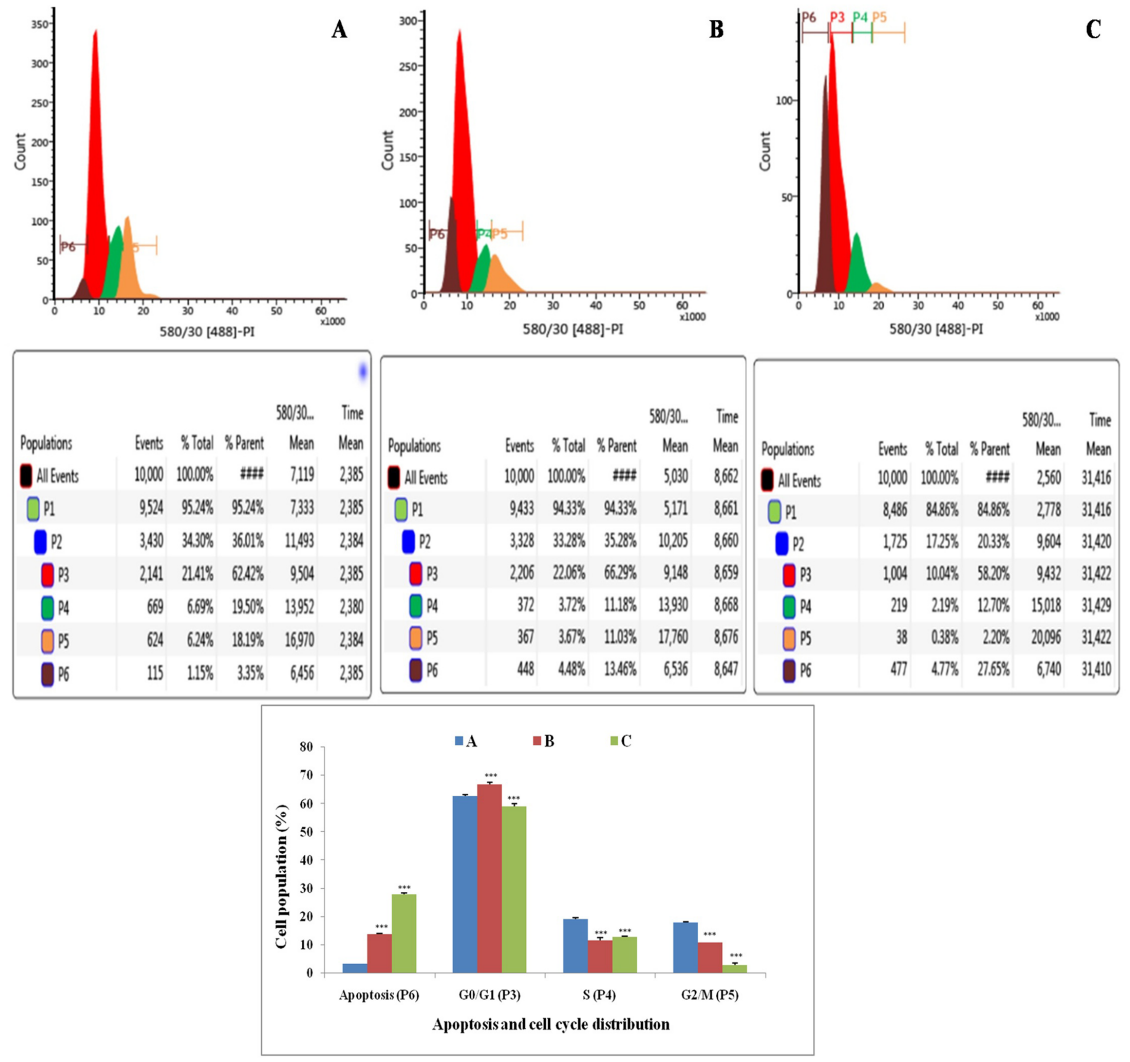
Effect of ALA on cell cycle arrest of ER+MCF-7 cells Flow cytometric analysis of cell cycle phase distribution was performed in control **(A)**; ALA (1/2 IC_50_) **(B)**; ALA (IC_50_) **(C)** after 18 h of treatment using PI staining. The histogram represents various content of DNA with actual number of cell present in three stages G0/G1 (P3), S (P4) and G2/M (P5). (X axis denotes fluorescence intensity of PE Texas red and Y axis denotes count). Values are presented as mean ± SD and the comparisons are made on the basis of one-way ANOVA followed by Bonferroni multiple test.

Flow cytometric analysis showed that treatment with ½ IC_50_ dose of ALA increased the DNA content by 1.20 fold than that of control in G0/G1 phase (P3). ALA also increased apoptotic cell burden by 2.07 fold in apoptotic phase (P6). ALA treatment was evident for the cell death in S (P4) and G2/M (P5), along with increase in the apoptotic cell burden by 7.86 fold. The results indicated that ALA treatment arrested the cell cycle in G2/M phase (Figure 4).

### Annexin-V FITC dot assay

**Figure 5:**
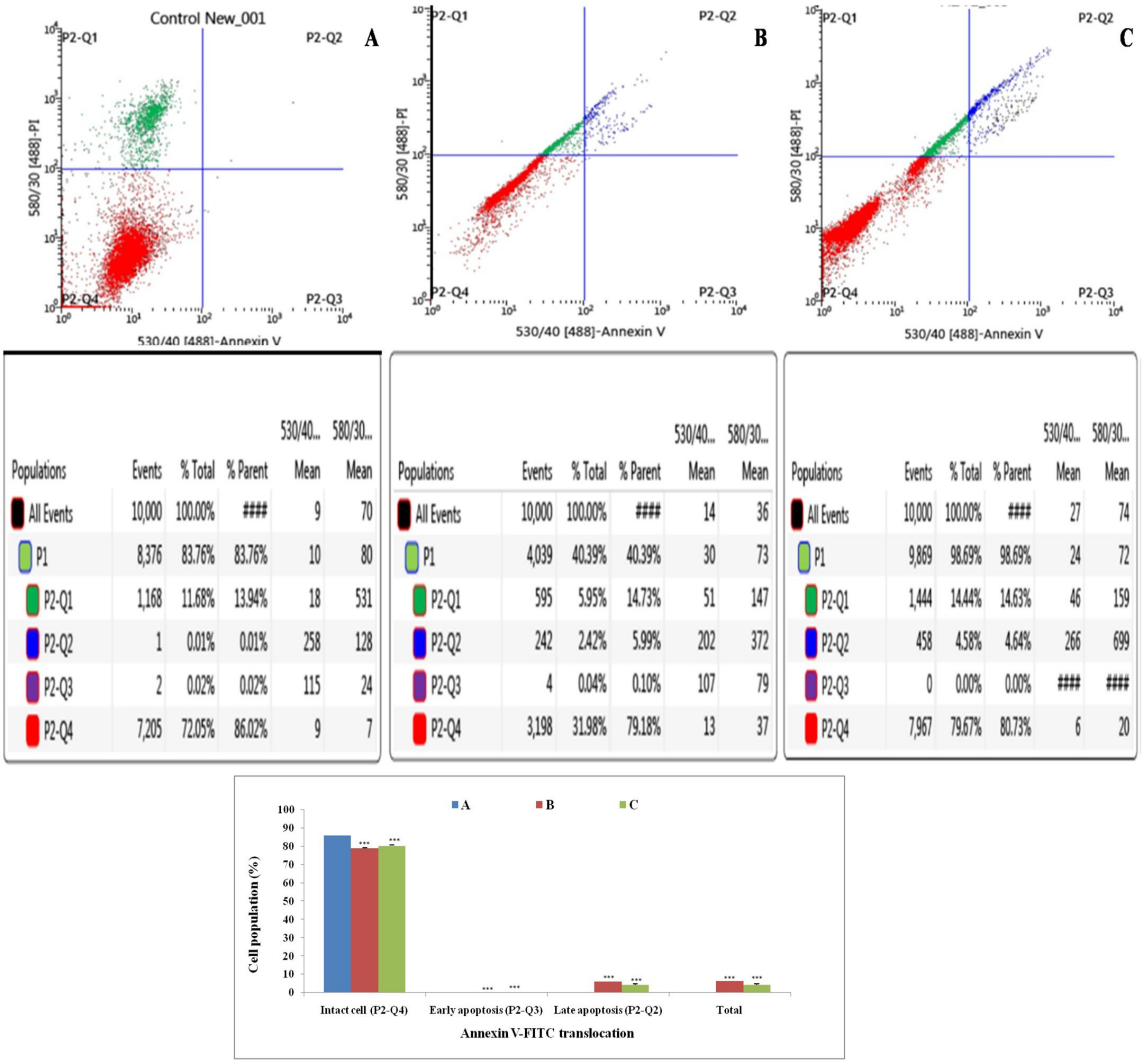
Effect of ALA on cell cycle arrest studied through Annexin-V FITC binding Study represent detection of cycle arrest in the ER+MCF-7 cells treated with control **(A)**; ALA (1/2 IC_50_) **(B)**; ALA (IC_50_) **(C)**. Cells were treated with Annexin V FITC and PI. Dual parameter dot plot of FITC-fluorescence (*x*-axis) vs. PI-fluorescence (*y*-axis) shows logarithmic intensity. Values are presented as mean ± SD and the comparisons are made on the basis of one-way ANOVA followed by Bonferroni multiple test.

Double labeling technique was used in flow cytometric analysis by using annexin-V FITC and PI. Lower left quadrant (Q1) is regarded as the population of live cells, lower right quadrant (Q2) is considered as the cell population at early apoptotic stage, upper right (Q3) quadrant represents the cell population at late apoptotic stage and upper left (Q4) quadrant is considered as necrotic cell population. Flow cytometric data analysis revealed that after 18 h of treatment with ½ IC_50_ and IC_50_ dose of ALA, ER+MCF-7 cells were in LR quadrant (Q2) in a dose dependent manner(4.58% against 2.42%) (Figure 5).

### Hemodynamic studies

Electrocardiogram (ECG) analysis revealed an increase in heart rate (HR) after 7, 12-Dimethylbenz (a) anthracene (DMBA) treatment (353.2±0.42beats/min). Treatment with ALA helped to restore the HR significantly close to normal (297.8±0.79 beats/min). No significant variability was recorded in the P wave duration after either of the treatments (Supplementary Figure 1, Supplementary Table 1). The heart rate variability (HRV) analysis of the ECG complex revealed the sharp decrease in the low frequency (LF) (9.24± 0.01ms^2^), high frequency (HF) (37.77± 0.05ms^2^) and very low frequency (VLF) (41.41±0.08 ms^2^) after the DMBA treatment. Treatment with ALA perceived dose-dependent restoration of the HRV parameters (Table 1).

**Table 1:** Effect of ALA on HRV changes in DMBA induced mammary gland carcinogenesis

	Control (0.9% normal saline, p.o)	Toxic control (DMBA 8 mg/kg i.v)	DMBA+ ALA (8 mg/kg i.v.+ 0.25ml/kg, p.o.)	DMBA+ALA (8 mg/kg i.v. + 0.5ml/kg, p.o.)
**Time Domain**				
**Average RR (ms)**	166.2±0. 02 ***	172.1±0.02	181.3±0.05***	165.5±0.01***
**Median RR (ms)**	166.6±0.06***	172.8±0.05	182.3±0.09***	165.5±0.09***
**SDRR (ms)**	6.25±0.01***	3.66±0.7	6.79±0.01***	5.63±0.01***
**CVRR**	0.02±0.01	0.02±0.01	0.12±0.02 ***	0.18±0.02***
				
**Frequency Domain**				
**LF (ms**^2^**)**	11.32±0.06***	9.24±0.01	14.02±0.04***	12.22±0.06***
**HF (ms**^2^**)**	39.70±0.09***	37.77±0.05	46.24±0.09***	43.30±0.07***
**LF/HF**	0.34±0.02	0.32±0.04	0.24±0.08	0.21± 0.1*
**VLF (ms**^2^**)**	42.13±0.07***	41.41±0.08	38.47±0.04***	42.87±0.09***

(Values are presented as Mean ± SD). Each group contains eight animals. Comparisons are made on the basis of the one-way ANOVA followed by Bonferroni multiple test. All groups are compared to the DMBA treated group (*p<0.05, **p<0.01, ***p<0.001).

### Morphological evaluation

#### Carmine staining of whole mount’s mammary gland

**Figure 6:**
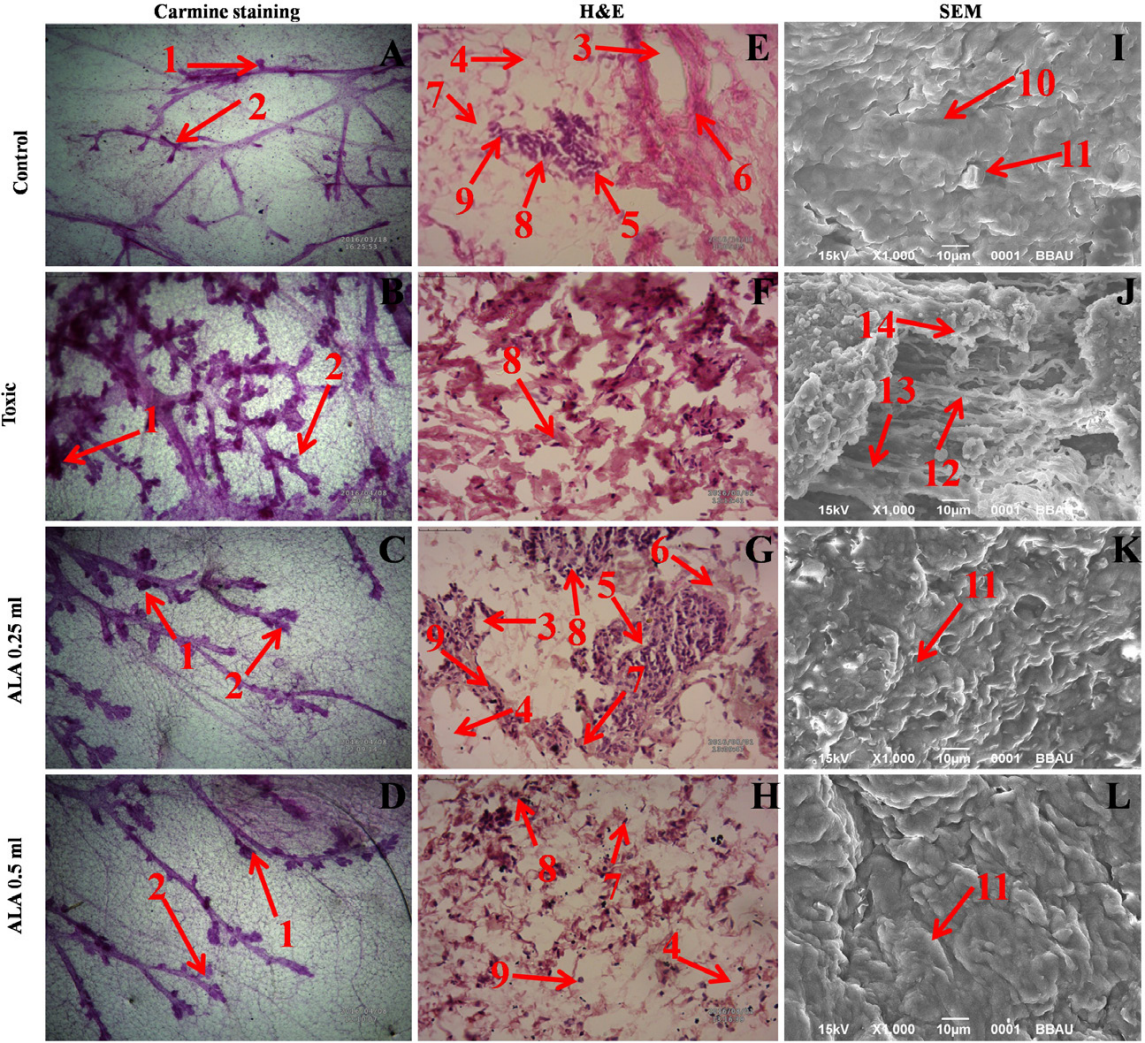
Microscopic evaluation of mammary gland tissue of the animal treated with ALA Whole mount carmine alum staining of ductal epithelium reveals the presence of lobules (1) and AB (2) **(A, B, C** and **D)**. The extent of AB and lobules formation was excessive in the DMBA treated group **(B)** which was subsided through respective treatment **(C** and **D)**. H&E staining of three respective groups **(E, G** and **H)** revealed duct (3), adipocytes (4), LCT (5), DCT (6), MEC (7), lymphocytes (8) and CEC (9) in control as well as ALA treated groups **(E, G** and **H)**. In DMBA treated group **(F)**, the cell morphology was distorted and cell organelles were absent. SEM analysis of control **(I)**, DMBA treated **(J)** and treatment group **(K** and **L)** revealed the differences in collagen layer (10), duct (11), small capillary network (12) and nodules (13) in respective groups.

In the DMBA treated group, there was marked increase in the lobules and alveolar buds (AB) count, representing cellular proliferation and ALA afforded a marked protection against the same. As a marker for growth and proliferation of the mammary gland tissue, ALA also decreased the differentiation score favorably (Figure 6A-6D).

#### Hematoxyline & Eosin staining of mammary gland tissue

The histopathological examination of the control tissue elaborated presence of duct; adipocytes; loose connective tissue (LCT); dense connective tissue (DCT); myoepithelial cells (MEC); lymphocytes and cuboidal epithelial cells (CEC) (Figure 6E). Treatment with DMBA recorded loss of duct, adipocytes, LCT, DCT and lymphocytes along with scattered CEC (Figure 6F). Infact, DMBA treatment distorted the histological architecture of the mammary gland tissue. Concomitant treatment with ALA imparted dose dependent restoration of the cellular architecture close to control (Figure 6G, 6H).

#### Scanning electron microscopy (SEM) of mammary gland tissue

The control tissue was evident for the features like intra-arterial cushion/collagenous covering, collagen layer and duct (Figure 6I). DMBA treatment evidenced loss of intra-arterial cushion (Figure 6J); development of small tumor micro-vessels and development of nodules (Figure 6J). Subsequent ALA administration perceived decrease in tumor micro-vessel formation, representing the deep impression of ALA on the branching sites along with restoration of intra-arterial cushion (Figure 6K, 6L).

### Antioxidant markers

Treatment with ALA validated the restoration of the antioxidant defense system in comparison to DMBA treated group. The protein and lipid peroxidation was very well evident after DMBA treatment. The ALA successfully decreased the level of protein carbonyl (PC) (32.95±0.9 nM/ml unit). Significant changes in the thiobarbituric acid reactive substances (TBARs) (0.21±0.02 nM of MDA/μg of protein) were also observed after ALA treatment. The level of glutathione (GSH) in DMBA treated group (1.03±0.09 mg %) was significantly restored after ALA treatment (1.19±0.01 mg %). Corresponding to the levels of superoxide dismutase (SOD) (0.037±0.01units of SOD/mg of protein) and catalase (13.91±0.97 nM of H2O2/ min/mg of protein) in DMBA treated group; ALA significantly accompanied to restore the same (i.e. 0.044±0.01units of SOD/mg of protein and 22.00±0.90 nM of H2O2/mg of protein) comparable to normal control (Table 2).

**Table 2:** Effect of ALA on oxidative stress markers against DMBA induced mammary gland carcinoma

Groups	TBARs (nM of MDA/μg of protein)	GSH (mg %)	SOD (Units of SOD/mg of protein)	Catalase (nM of H_2_O_2_/ min/mg of protein)	Protein carbonyl (nM/ml unit)
Control (0.9% normal saline, p.o.)	0.15±0.03***	1.10±0.06***	0.042±0.01	18.45±0.3***	40.43±0.11***
Toxic control (DMBA 8 mg/kg, i.v.)	0.30±0.02	1.03±0.09	0.037±0.01	13.91±0.97	45.57±0.92
DMBA +ALA (8 mg/kg, i.v. + 0.25 ml/kg, p.o.)	0.23±0.19***	1.11±0.02***	0.042±0.02	19.60±0.03***	34.42±0.68***
DMBA +ALA (8 mg/kg, i.v. + 0.5 ml/kg, p.o.)	0.21±0.02***	1.19±0.01***	0.044±0.01	22.00±0.90***	32.95±0.9***

(Values are Mean ± SD), each group contains eight animals. Comparisons are made on the basis of one-way Anova followed by Bonferroni test. All groups are compared to the DMBA treated group (*p<0.05, **p<0.01, ***p<0.001).

### ^1^H-NMR method for serum metabolites profiling

**Figure 7:**
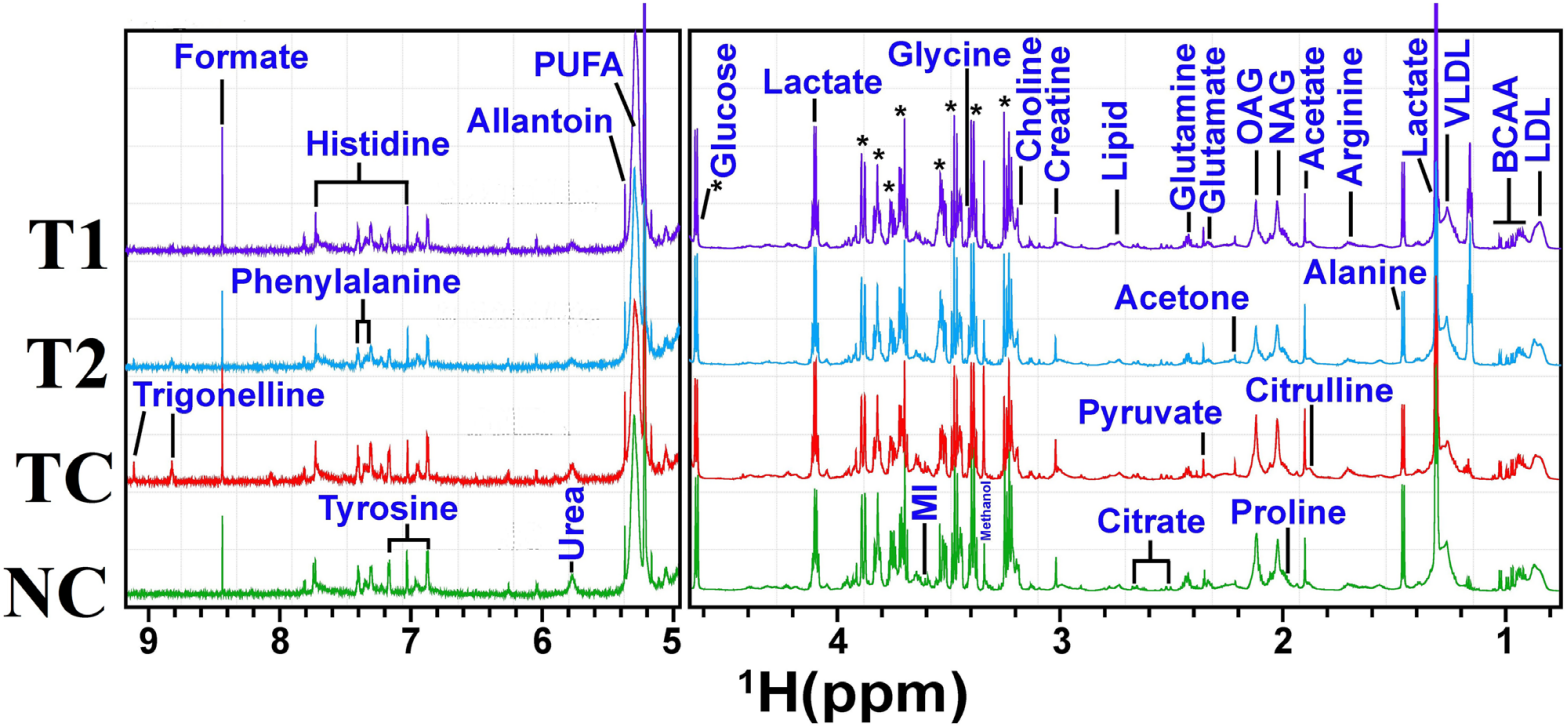
Stack plot of representative 1D ^1^H NMR spectra of rat sera obtained from different groups The representative1D ^1^H CPMG NMR spectra of rat serum obtained from different groups. The peaks annotated in the figure shows the assignments of serum metabolites. Groups were differentiated as: normal control (NC), toxic control -DMBA (TC), ALA-0.25 ml/kg (T1) and ALA-0.5 ml/kg (T2). The abbreviations used are LDL/VLDL: low/very-low-density lipoproteins; PUFA: polyunsaturated fatty acids; BCAA: branched chain amino acids: isoleucine, leucine, valine; MI: myo-inositol, OAG: O-acetyl glycoproteins; NAG: N-acetyl glycoproteins.

A typical ^1^H CPMG NMR spectra of serum samples obtained from different groups is shown in Figure 7. The NMR spectra showed signals, mainly from lipids/lipoproteins [(e.g. low density lipoprotein (LDL), very low density lipoprotein (VLDL), PUFAs etc.)], membrane metabolites [(e.g. choline, phosphocholine (PC), and glycerophosphocholine (GPC)], N-acetyl and O-acetyl glycoproteins (NAG, OAG), and amino acids [(e.g. leucine, isoleucine, valine, alanine, lysine, proline, glutamine, glutamate, histidine, tyrosine, and phenylalanine etc.)]. Other identified metabolites were, glucose, lactate, acetate, citrate, creatine and allantoin.

#### Multivariate statistical analysis

**Figure 8:**
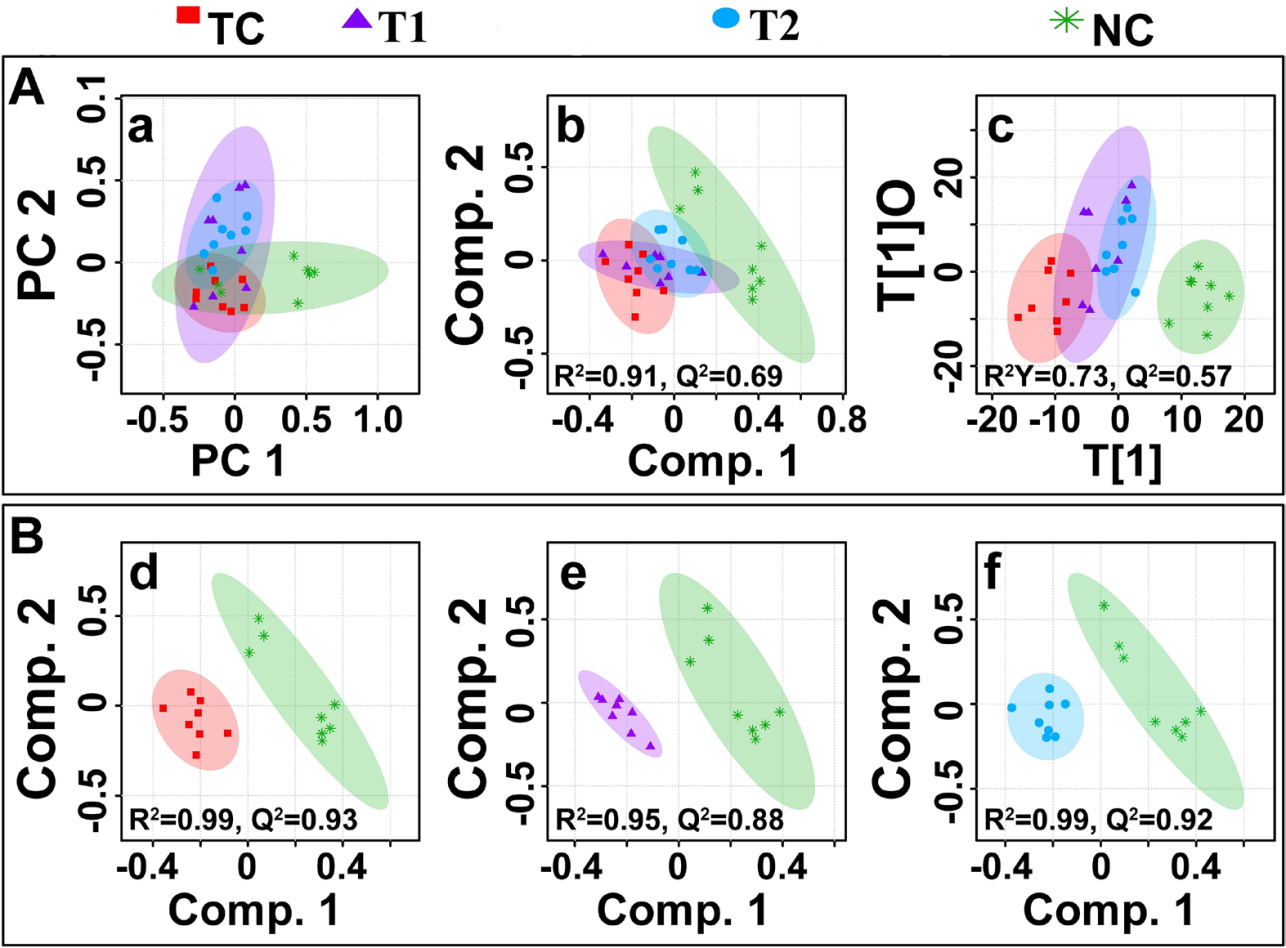
Multivariate analysis **(A)** The combined2D PCA **(a)** and 2D PLS-DA **(b)** 2D OPLS-DA **(c)** score plots derived from cumulative analysis of 1D ^1^H CPMG NMR spectra comprising of all the groups: normal control (NC), toxic control -DMBA (TC), ALA-0.25 ml/kg (T1) and ALA-0.5 ml/kg (T2). Color circles indicate the 95% confidence interval for each class **(B)**. The pairwise PLS-DA score plots **(d-f)**: **(d)** between normal control (NC) and DMBA treated toxic control (TC) group, **(e)** between NC and DMBA+0.25ml/kg-ALA, and **(f)** between NC vs DMBA+ALA-0.5ml/kg.

The multivariate data analysis was performed to find out specific metabolic changes induced by DMBA treatment and to further reveal the effect of ALA treatment on these metabolic alterations. The ^1^H-NMR dataset was analyzed using standard multivariate analysis methods including principal component analysis (PCA), partial least square discriminant analysis (PLS-DA), and OPLS-DA to study trends and show clusters among the groups (Figure 8A). First, unsupervised PCA score plots were constructed for an initial overview of the data set and identify the outlier samples (Figure 8a). The majority samples were located in 95% confidence interval. Therefore, all of the samples were used in the analysis to ensure the maximum information. Supervised PLS-DA score plots were generated to further improve the separation between the four groups. PLS-DA score plots showed that ALA treated groups were well separated from the normal control group, with a significantly higher quality of fit and predictability (i.e. R2, Q2> 0.5, Figure 8b). Next, to minimize the possible contribution of intergroup variability and to improve the group discriminatory features, OPLS-DA was performed. As shown in Figure 8c, the OPLS-DA score plots depicted excellent grouping of samples within each group and improved separation for the different treatments with significantly well explained variation and predictive capability (i.e. R2Y, Q2> 0.5, Figure 8c). The OPLS-DA score plot analysis further revealed that ALA (0.25ml/kg) and ALA (0.5ml/kg) treatments are progressively mitigating the toxic effect of DMBA treatment as evident by the shifting of these groups within the control group.

To further reveal the biochemical effects produced by DMBA treatment and ascertain if ALA treatment could reset back these changes, the pairwise PLS-DA analysis with respect to normal control rats was performed. Figure 8B shows the 2D score plots derived from PLS-DA analysis in each case (the corresponding PCA score plots are shown in Supplementary Figure 2). The quality and reliability parameters (R2 and Q2) assessed for each paired PLS-DA model (Figure 8B) were found to satisfactorily higher (R2, Q2>0.5) suggesting that PLS-DA can be employed to evaluate the biochemical effects of ALA treatment. Discriminatory (PLS-DA) analysis between DMBA treated, and normal control groups revealed twenty five metabolite entities significantly perturbed in DMBA treated group with respect to control, using VIP score >1 for discrimination significance (Supplementary Figure 3).

**Figure 9:**
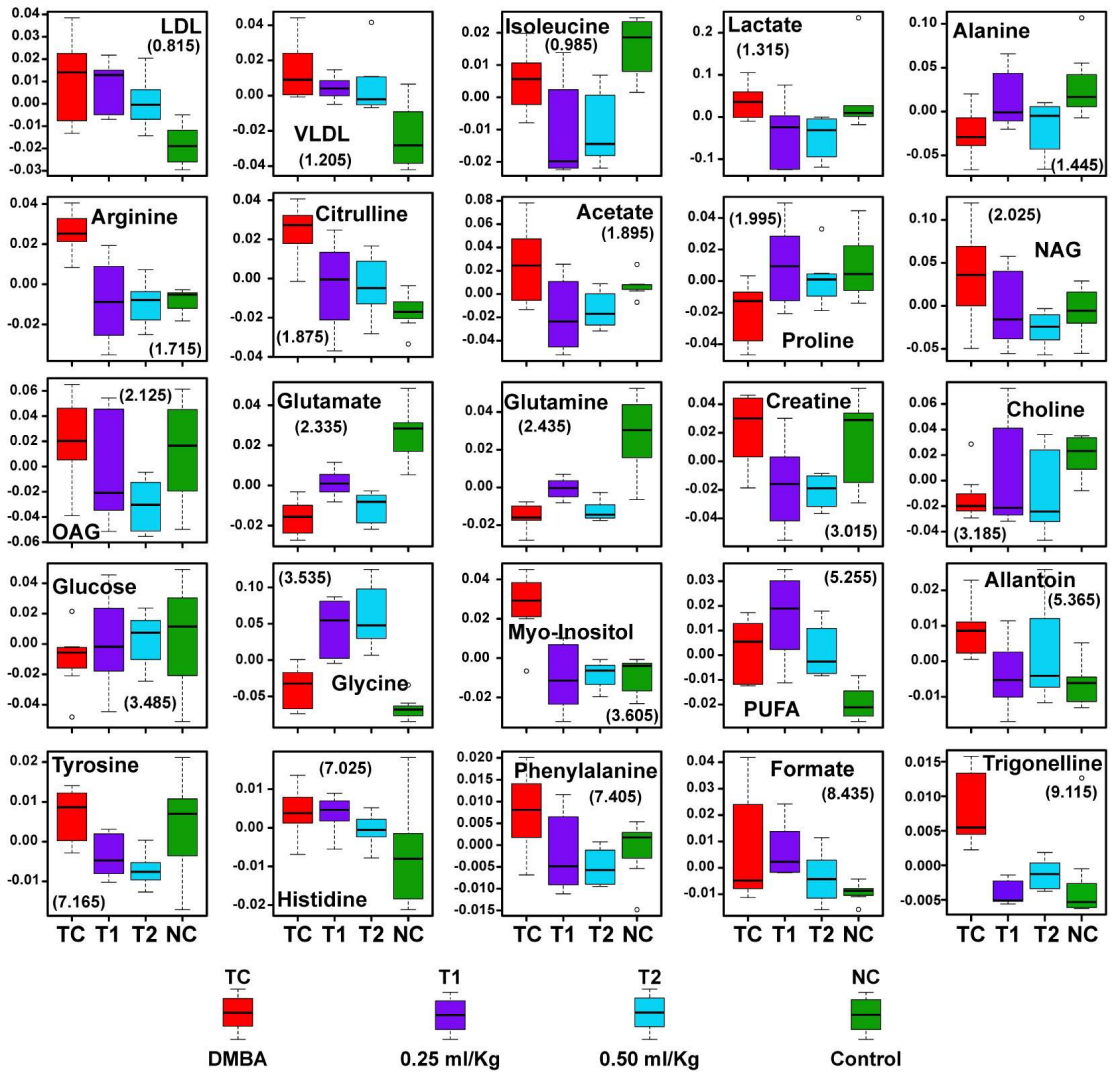
Biochemical effects of ALA treatment Representative box-cum-whisker plots showing quantitative variations of relative signal integrals for serum metabolites relevant in the context of pathophysiology of mammary gland cancer. For presented metabolite entities, the VIP score >1 and statistical significance is at the level of p ≤ 0.05. In the box plots, the boxes denote interquartile ranges, horizontal line inside the box denote the median, and bottom and top boundaries of boxes are 25^th^ and 75^th^ percentiles, respectively. Lower and upper whiskers are 5^th^ and 95^th^ percentiles, respectively.

As listed in Table 3, twenty five metabolites were found to be significantly perturbed in the sera of DMBA treated rats compared with normal control rats. DMBA treated rats had elevated levels of lipids, VLDL/LDL lipoprotein, lactate, NAG, OAG, PUFA, arginine, citrulline, creatine, myo-inositol, glycine, allantoin, tyrosine, histidine, phenylalanine, formate and trigonelline in their sera. Moreover, decreased levels of glucose, choline/GPC, and several amino acids including, alanine, isoleucine, valine, glutamate and glutamine was recorded as well. Further, we found that the metabolic alterations which were observed in DMBA treated group were ameliorated after the ALA treatment as evident from the box plot (Figure 9).

**Table 3:** Metabolic variabilities among the groups treated with DMBA and ALA when compared to normal control

#	^1^H ppm	Metabolite (↓↑)	Control vs		
			DMBA	ALA-0.25	ALA-0.5
1	0.825-0.885	LDL	*(↑)	*(↑↑)	*(↑↑↑)
2	1.205-1.235	VLDL	*(↑)	*(↑↑)	*(↑↑↑)
3	5.245-5.355	PUFA	*(↑)	*(↑)	*(↑↑↑)
4	0.985	Isoleucine	(↓)#	*(↓↓)	(↓↓↓)#
5	7.025, 7.725	Histidine	*(↑)	*(↑↑)	*(↑↑↑)
6	7.405	Phenylalanine	*(↑)	(↓↓)#	*(↓↓)
7	1.455	Alanine	*(↓)	(↓↓)#	*(↓↓)
8	1.695-1.715	Arginine	*(↑)	(↓↓)#	(↓↓)#
9	2.435	Glutamine	*(↓)	*(↓↓↓)	*(↓↓)
10	1.995	Proline	*(↓)	(↑)	*(↓↓↓)
11	3.535	Glycine	*(↑)	*(↑)	*(↑)
12	6.885, 7.175	Tyrosine	(↑)#	*(↓↓↓)	*(↓↓)
13	1.865-1.885	Citrulline	*(↑)	(↑↑)#	(↑↑↑)#
14	3.015	Creatine	(↑)	*(↓↓)	*(↓↓)
15	1.895	Acetate	(↑)#	*(↓↓)	*(↓↓↓)
16	2.025	NAG	(↑)	*(↓↓↓)	*(↓↓)
17	2.115, 2.125	OAG	(↑)#	*(↓↓)	*(↓↓)
18	2.325	Glutamate	*(↓)	*(↓↓↓)	*(↓↓)
19	3.885, 5.215	Glucose	*(↓)	*(↓)	*(↓)
20	3.605	Myo-Inositol	*(↑)	(↓↓)#	(↓↓↓)
21	1.315	Lactate	(↑)	*(↓↓)	*(↓↓)
22	3.185	Choline	*(↓)	(↓↓)#	*(↓↓)
23	5.365	Allantoin	*(↑)	(↑)#	*(↑)
24	8.435	Formate	*(↑)	*(↑↑)	(↑↑↑)#
25	8.825, 9.115	Trigonelline	*(↑)	(↑↑↑)#	(↑↑)#

The up and down arrows represent, respectively, increased and decreased metabolite levels. A ↑↑↑/↓↓↓ or ↑↑/↓↓ score was given to the metabolites of the treatment dose which showed ameliorating effects from DMBA towards control. Abbreviations used are as follows: LDL (low density lipoproteins); VLDL (very low density lipoproteins); NAG (N-acetyl glycoprotein); OAG (O-acetyl glycoprotein); PUFA (poly unsaturated fatty acids).

Note-: Symbols * = p-value < 0.05, # = VIP Score < 1.

### Assay for caspase 3 and caspase 8

**Figure 10:**
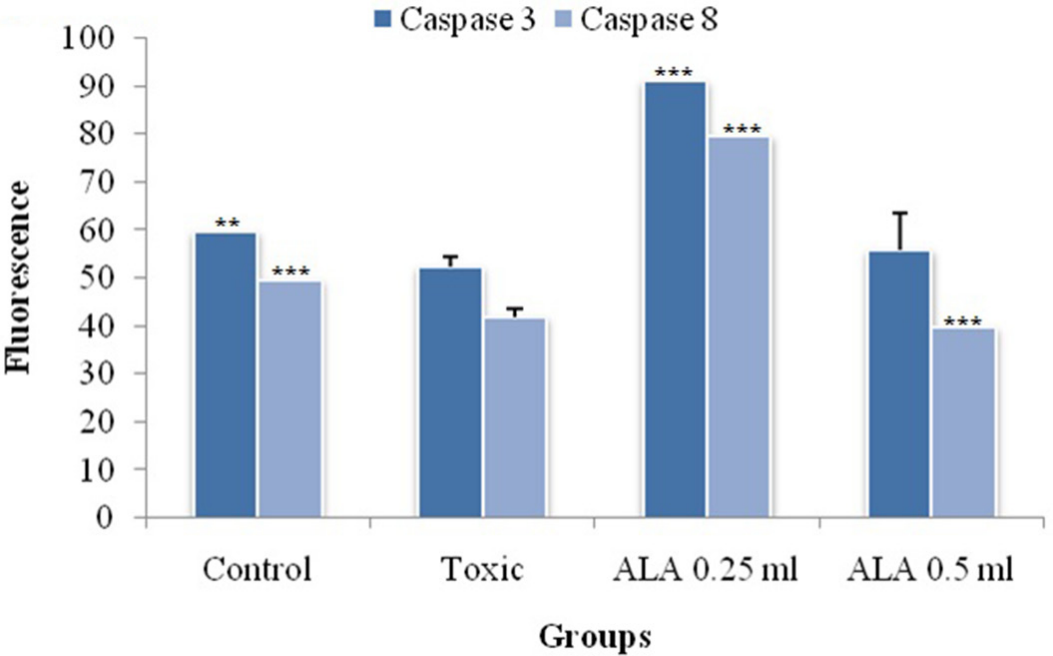
Effect of ALA on caspase3 and caspase8 The activity of caspase was detected by commercial fluorescence based assay. Data are expressed as mean+ SD of individual groups. Comparisons are made by the one-way ANOVA followed by Bonferroni multiple test. All groups are compared to the DMBA treated group (*p<0.05, **p<0.01, ***p<0.001).

ALA (0.25 ml/kg) increased the levels of caspase 3 and caspase 8 in the DMBA treated animals significantly (Figure 10).

### Western blotting

**Figure 11:**
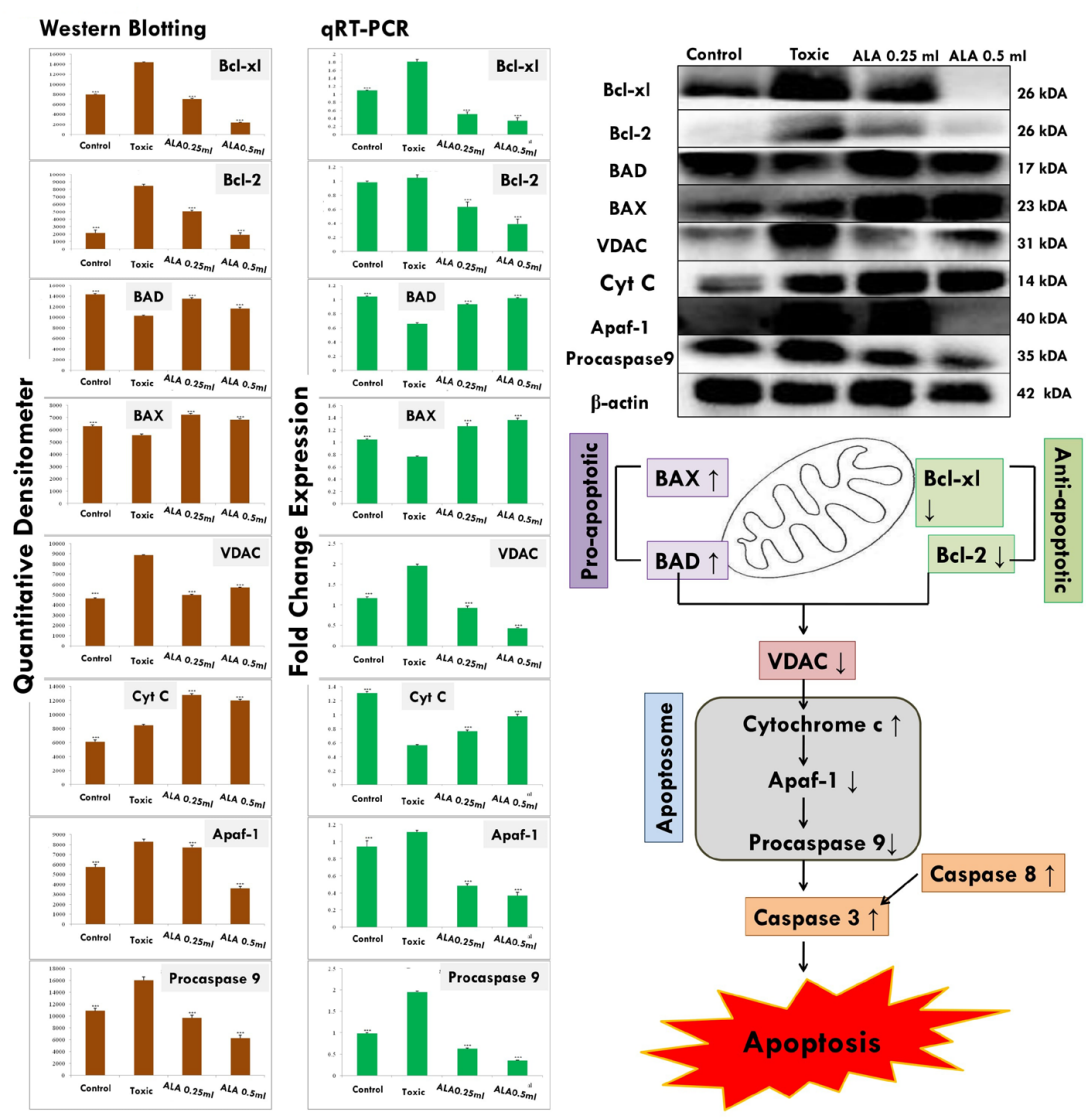
ALA mediated activation of mitochondrial associated protein signaling in mammary gland cells Protein extracted from individual groups [1-control, 2-DMBA treated, 3-ALA (0.25 ml/kg, p.o. + DMBA 8 mg/kg, i.v.) and 4- ALA (0.5 ml/kg, p.o. + DMBA 8 mg/kg, i.v.)] were subjected to immunoblotting of proapoptotic (BAX) and anti-apoptotic (Bcl-2 and Bcl-xl) protein with downstream apoptotic markers (VDAC, cytochrome-c, Apaf-1and procaspase9) of respective pathway. mRNA expression of above mentioned protein were also in line with the findings of immunoblotting assay. β-actin was used as loading control. Each experiment was performed in triplicate. Values are presented as mean ± SD. Comparisons are made by the one-way ANOVA followed by Bonferroni multiple test. All groups are compared to the DMBA treated group (*p<0.05, **p<0.01, ***p<0.001).

**Figure 12:**
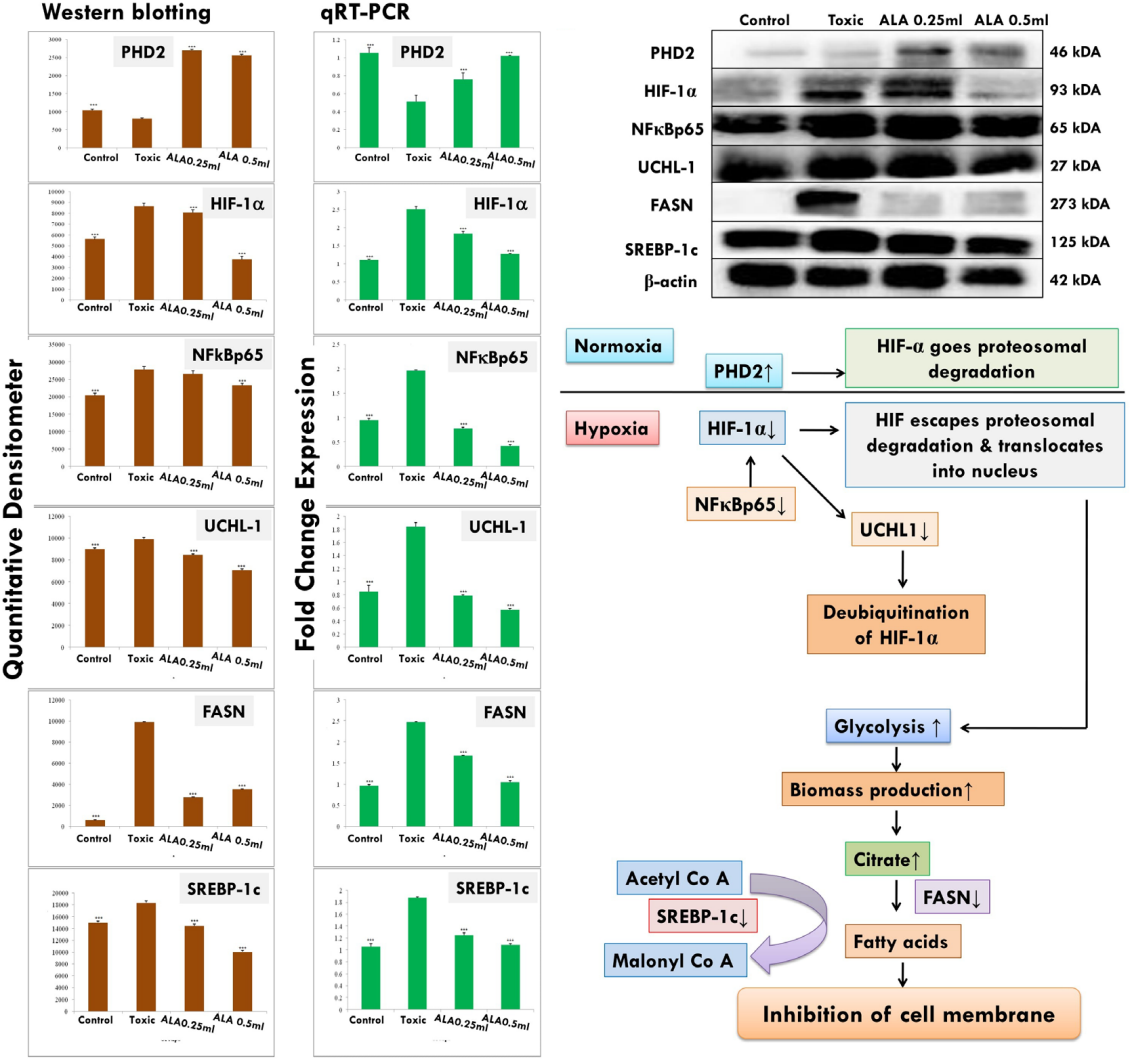
Effect of ALA on HIF-1α mediated hypoxic markers and fatty acid synthesis regulators Immunoblotting of respective individual group [1-control, 2-DMBA treated, 3-ALA (0.25 ml/kg, p.o. + DMBA 8 mg/kg, i.v.) and 4- ALA (0.5 ml/kg, p.o. + DMBA 8 mg/kg, i.v.)] for HIF-1α, PHD2, and FASN conclude the hypoxic microenvironment after DMBA treatment. Perspective correlations of NFκBp65, UCHL-1 and SREBP-1c with the above mentioned protein are well defined through the experimental pathway. Excised mammary gland tissue sample lysed in trizol for RNA extraction and analyzed for the mRNA expression of HIF-1α, PHD2, FASN, NFκBp65, UCHL-1 and SREBP-1c by qRT-PCR: fold induction is relative to tissue under hypoxic conditions after normalization to the β-actin expression. Each experiment was performed in triplicate. Values are presented as mean ± SD. Comparisons are made on the basis of the one-way ANOVA followed by Bonferroni multiple test. All groups are compared to the DMBA treated group (*p<0.05, **p<0.01, ***p<0.001).

The expression of anti-apoptotic proteins (Bcl-2 and Bcl-xl) was increased after DMBA administration with vice versa effect upon pro-apoptotic markers (BAD and BAX). Treatment with ALA helped to restore the anti-apoptotic and pro-apoptotic markers favorably suggesting apoptosis. When perceived through the downstream markers of mitochondrial mediated apoptosis (VADC, cytochrome c, Apaf-1 and pro-caspase 9), the DMBA afforded increased expression of VDAC, Apaf-1 and pro-caspase9 along with curtailment of cytochrome c expression (Figure 11). Treatment with ALA afforded marked regulation of apoptotic markers favoring apoptosis. Treatment with DMBA also afforded commendable hypoxia as perceived through increased expression of NFκBp65, UCHL-1, HIF-1α, FASN and SREBP-1c; and decreased expression of PHD2. Concomitant ALA treatment afforded abatement of hypoxic markers significantly (Figure 12).

### qRT-PCR

The genetic phenotypes for the protein markers for mitochondrial death pathway were validated through qRT-PCR assay (Figure 11). The findings from the immunoblotting assay were endorsed by the qRT-PCR studies, embarking that the effect of ALA is mediated through regulating the respective genetic phenotypes. The qRT-PCR studies for the hypoxic markers recorded similar pattern of fold changes as perceived through the immunoblotting assay (Figure 12).

## DISCUSSION

The *in-vitro* studies affirmed significant cytotoxic and apoptotic potential of ALA against ER+ MCF-7 cells, when scrutinized through MTT assay (Figure 1) and AO/EtBr staining. The ALA treated AO/EtBr stained cells were evident for the presence of apoptosis as visualized with nuclear shrinkage, chromatin condensation, fragmented nuclei, and membrane blebbing [11] (Figure 2). Considering the fact that mitochondria participates in apoptosis, the effect of ALA on mitochondrial membrane potential was validated through cationic dye JC-1. Greater membrane potential results in higher accumulation of JC-1 and formation of J-aggregates, which exhibit maximal fluorescence emission at ∼590 nm (red)[12]. Loss in mitochondrial membrane potential results in failure to accumulate J-aggregate and their existence in the monomeric form, which exhibit green fluorescence. Therefore, JC-1 is considered to be an indicator of mitochondrial potential and decrease in mitochondrial membrane potential is an indicative of apoptosis as perceived after the ALA treatment (Figure 3). Subsequent studies affirmed the cell cycle arrest in G2/M phase by the ALA treatment (Figure 4). Translocation of phosphatidylserine (PS) to the outer leaflet of the cellular membrane is a key marker of apoptosis and could be validated through Annexin-V, (calcium-dependent phospholipid binding protein) labeled with FITC and PI [13]. The same revealed, ALA mediated apoptosis to be associated with PS translocation (Figure 5). All in all, *in-vitro* studies affirmed participation of two major hallmarks of apoptosis, including changes in mitochondrial permeability and PS translocation after the ALA treatment in a dose dependent manner.

As encouraged through *in-vitro* studies, the efficacy of ALA was further validated *in-vivo* against DMBA induced mammary gland carcinoma. DMBA is a polycyclic aromatic hydrocarbon and is used to induce mammary gland carcinoma in experimental animals. The mammary tumors produced from DMBA are morphologically and histopathologically very similar to human tumors [14]. DMBA is an indirect carcinogen, and requires metabolic activation by cytochrome P450 enzymes to reactive metabolites, i.e. dihydrodiolepoxides and forms mutagenic DNA adduct [15]. Mainly two enzymes viz cytochrome P4501B1 (CYP1B1) and microsomal epoxide hydrolase (EPHX1) are responsible for DMBA bioactivation [16].

Autonomic dysfunction associated with cardiovascular complications, poor quality of life and premature mortality are well reported phenomenon in the breast cancer survivors [17]. Infact, autonomic dysfunction is now a day is considered as a non-invasive prognostic marker for chemotherapeutic regime [18]. HRV is a clinical marker of autonomic function and cancer patients with low HRV are associated with increased cardiovascular risk [19]. Treatment with ALA helped to restore the autonomic control and therefore, could be designated to have good prognostic value in chemoprevention regime as proposed through ALA (Table 1, Supplementary Table 1 and Supplementary Figure 1). It would be appropriate to put on records, that low dose of ALA imparted more favorable regulation of ECG and HRV paradigms in comparison to high dose of ALA. Interestingly, high dose of ALA further deteriorates time and frequency domain parameters of HRV. Authors would also like to submit that a meta-analysis concluded that short term PUFA’s supplementation may favorably influence HRV parameters as observed through low dose of ALA in the present study [20].

Cellular proliferation and angiogenesis are well-defined hallmarks for cancer progression [21]. As an advantage of *in-vivo* studies, same could be studied through carmine staining, H&E staining and SEM. The carmine staining was evident for the development of proliferative lesions as represented by increase in AB in DMBA treatment. The AB represents the largest bulbous structure located at the distal end of the mammary epithelial tree and is the site for the malignant transformation [22]. The DMBA treatment was evident for increase in AB, which is in corroboration with a previous study [23] (Figure 6A-6D). Treatment with DMBA was also evident for the scattered pattern of CEC, hardly located LCT and DCT along with loss of duct and MEC, as visualized by H&E staining (Figure 6E-6H). Marked cellular proliferation after the DMBA treatment was also evident for increase in micro vessel formation, loss of intra-arterial cushion and vascular conglomeration when perceived through SEM analysis (Figure 6I-6L). It would be appropriate to remark, that findings derived from the carmine staining, H&E staining and SEM analysis are well in line with previous reports [24, 25]. Treatment with ALA demarcated a marked impression on cellular architecture and morphology, as evident through decrease in AB, restoration of the cellular architecture, decrease in micro vessel formation with profound impression on the branching sites. Enlarged capillaries, which are the sign of rapidly growing tumors were also absent in ALA treated groups. All in all, ALA imparted a significant dose-independent effect to curtail cellular proliferation and therefore, warrant’s further validation through biochemical and molecular biology markers.

The biochemical markers could be majorly categorized as the ones associated with antioxidant defense or to the physiological mechanisms, and authors validated both. Reactive oxygen species (ROS) are constantly produced in all aerobic cells and are counter balanced by the antioxidant enzymatic defense [26]. However, during anaerobic/hypoxic conditions like cancer (due to increased cellular proliferation), the counter balance effects of antioxidant enzymes are subsided [27]. The damage to the cellular lipids and proteins can be validated through increased production of TBARs and PC respectively; which was very well evident after the DMBA treatment. The increased ROS production also inhibits the enzymatic antioxidant defense of GSH, SOD and catalase, as they all work in tandem to curtail ROS through series of peroxidation, dismutation and oxidation reactions [28]. The decrease in the enzymatic defense of SOD, catalase and GSH, suggest their increased utilization, which was profoundly evident after the DMBA treatment. It would be appropriate to remark that ALA administration curtailed the levels of TBARs and PC with restoration of enzymatic antioxidant defense of SOD, catalase and GSH (Table 2). In contradiction to the findings in the preceding paragraphs, ALA high dose was observed to be more efficacious in comparison to low dose and the same could be attributed to the fact that ALA being a PUFA would have rendered himself against the ROS (in lieu of cellular lipids) and acted as a pro-oxidant (dose dependently) rather than anti-oxidant as elaborated by our laboratory in a previous report [29].

The DMBA induced changes indicated that subsets of metabolites were changed in similar way in all groups, which were ameliorated by ALA treatment. The decreased glucose and increased lactate and myo-inositol levels in DMBA are well correlated with warburg effect and increased bioenergetics demand for cellular proliferation [30] (increased glycolytic activity with increased lactate production)[31]. Further, significant increase in the levels of lipoproteins (LDL/VLDL) and PUFAs, in DMBA treated animals suggests increased requirement of the building blocks of the cell membranes in rapidly proliferating tumor cells [32]. Exogenous ALA supplementation provided further increase in the levels of lipoproteins (LDL/VLDL) and PUFA’s and the same could be attributed to the fact that ALA is a polyunsaturated fat. The lower levels for choline could be the consequence of excessive need for choline and its derivatives during rapid cell proliferation [33, 34].

Inflammation is a most common clinical manifestation of various cancer types and triggers a hyper-catabolic state, resulting in increased energy requirements. Consistent with this phenomenon the increased concentrations of serum acetyl-glycoproteins (both NAG and OAG) (acute phase anti-inflammatory proteins expressed during inflammation and immune response) was recorded after the DMBA treatment and is in line with previous investigations in liver disease, inflammatory disease, and cancer [35–37]. As expected, ALA (an anti-inflammatory PUFA) curtailed the expression of acute phase proteins expressed during inflammation [38].

The increased levels of amino acids like arginine, glycine, histidine, tyrosine, creatine and phenylalanine indicate abnormal/aberrant biosynthesis of amino acids in DMBA treated rats. The increased levels of arginine and citrulline suggest protein catabolism, deriving overall picture of high metabolic activity, a hallmark for tumor progression [39],[40],[41]. The high metabolic activity as evident through increased levels of amino acids was also endorsed through increased levels of formate (a product of glycine metabolism through glycine succinate pathway) [42]. The decreased level of glucogenic amino acids (glutamate, glutamine, proline, isoleucine and alanine) in DMBA treated group suggests their increased utilization in energy production. Proline metabolism is especially important in nutrient stress, as it is interchangeably converted into glutamate and glutamine. Concomitant ALA treatment further diminished the levels of glucogenic amines which could be accounted to the fact that exogenous ALA would have provided a faulty lipid to the fastly growing tumour cells. Whereas the requirement for the amino acids as a building block for cellular membranes was prevalent till such time.

The deregulated metabolites represent altered cancer cell energy metabolism including amino acid metabolism (glutamate, glutamine, alanine, etc.), glycolysis or gluconeogenesis (glucose, and lactate,) and lipid metabolism (LDL, VLDL, choline, and acetate) and are associated with high rate of glycolysis. Most of the metabolic changes in DMBA treated animals were reset back to normal after ALA administration, suggesting that the ALA has potential to balance the metabolic abnormalities in fastly growing cells (Figure 9).

The findings from the antecedent sections are clear indicative that ALA has a definite say towards mitochondrial energy production, glycolysis and fatty acid synthesis to fuel the high energy and biomass demands for the tumor growth. Given the same, we considered it worth to validate the molecular biology markers for mitochondrial apoptosis and fatty acid synthesis.

The mitochondrial apoptosis is governed through series of pro-apoptotic and anti-apoptotic regulators [43]. The ALA decreased the expression of anti-apoptotic (Bcl-2 and Bcl-xl) along with increase in pro-apoptotic (BAX and BAD) markers. The mitochondrial apoptotic changes are further associated with decreased expression of VDAC, due to loss in the channel integrity with concomitant release of cytochrome c [44, 45]. The immunoblotting and qRT-PCR studies affirmed the decreased expression of VDAC with concomitant increase in cytochrome c expression after the ALA treatment. Once released from mitochondria, cytochrome c binds with Apaf-1 and procaspase 9 to form adduct termed, apoptosome [46]. Apoptosome formation accounts for the decreased cytosolic levels of Apaf-1 and procaspase 9 and the same was evident after the ALA treatment [47]. Apoptosome formation further cleaves the procaspase 9 to give active caspase 9 which is involved in the intrinsic apoptotic pathway and mediate the activation of effecter caspase 3 and 8 for execution of apoptosis [48]. Treatment with ALA (low dose) increased the level of caspase 3 and 8 and thereby accredited apoptosis. It would be appropriate to pendown that ALA (high dose) was not as effective as low dose, which is not in line with the findings reported in preceding paragraphs. However, authors are opined that such observation could be the consequence of feedback inhibition of caspase 3 and 8 [49]. Similar type of findings with other PUFA’s, particularly DHA has been reported previously [50, 51]. From the above line of evidences, authors could easily derive that the proliferative and angiogenic effects of DMBA are curtailed by ALA through activating the mitochondrial-mediated apoptosis death pathway (Figures 10 and 11).

Rapidly growing tumor cells derive energy majorly through glycolysis due to insufficient oxygen (hypoxia)[52]. The hypoxia in the cellular microenvironment is controlled with the help of hypoxia inducible factor-1α (HIF-1α), which is further synchronized through oxoglutarate dependent hydroxylase called prolyl hydroxylase -2 (PHD2)[53]. Increased glycolytic activity in the rapidly proliferating cells is coupled with increased FASN to meet the fatty acid requirements through de novo synthesis [54]. ALA treatment increased the PHD2 expression and curtailed the HIF-1α expression, once validated through immunoblotting and qRT-PCR assay. The curtailment of HIF-1α was also validated through reduced expression for NFκBp65 and UCHL-1, as NFκBp65 impart positive modulatory effect upon HIF-1 α and UCHL-1 felicitates the deubiquination of HIF-1 α (HIF-1 α to escape proteasomal degradation during hypoxia through deubiquination)[55, 56]. ALA treatment decreased the FASN expression at protein and mRNA level, suggesting decreased de novo synthesis of fatty acids (Figure 12). Decreased fatty acid synthesis was further endorsed through abated expression for SREBP-1c, a regulator for conversion of acetyl CoA to malonyl CoA during de novo fatty acid synthesis [57, 58].

Supplementation with ω-3 PUFA can impart favorable modification of membrane fatty acid composition or detrimental oxidative metabolism within the cell. Low dose of ω-3 PUFA has been reported not to induce harmful modifications of oxidative cell metabolism, as modifications of membrane fatty acid composition occur [59, 60]. The reports also suggest the possibility for use/better efficacy of low doses of ω-3 PUFA for chemoprevention and same was observed after the ALA treatment [61, 62]. On the whole, ALA curtailed the hypoxic microenvironment, de novo fatty acid synthesis and arbitrated mitochondrial mediated apoptosis to restrain proliferative and angiogenic effects of DMBA.

## MATERIALS AND METHODS

### Drugs and chemicals

ALA (463-40-1) (0.914gm/ml) was purchased from TCI Chemicals (India) Pvt. Ltd. RPMI 1640 (Gibco, USA); fetal bovine serum (FBS) (Gibco-10270); trypsin (Gibco, USA); eagle balanced salt solution (EBSS)(Gibco, 2018-11); hank’s balanced salt solution (HBSS) (Himedia, TL1190); EtBr (Himedia, MB071); AO(Himedia, MB116); JC-1 assay kit (Thermo Scientific, M34152); PI (SC-3541); tamoxifen citrate (TMX) (Tammodex 20, Biochem Pharmaceuticals India); penicillin- streptomycin (Thermo Scientific, 15410-163); gentamycin (Thermo Scientific, 15710-049);3-(4, 5-Dimethyl-2-thiazolyl)-2, 5-diphenyl-2H-tetrazolium bromide (MTT) (Himedia, TC191); RNase (SRL, 58895); dimethyl sulfoxide (DMSO) (Merck, 1.16743.0521); DMBA (Sigma Aldrich, 57-97-6); ponceau S (Himedia, ML045); sodium cacodylate (Sigma Aldrich, C0250); collagenase type 4 (Himedia, TC-214); hyaluronidase (Himedia, TC331); hematoxylin (Himedia, S058); eosin (Himedia, S007); RIPA lysis buffer (Amresco, N653); protein assay kit (Amresco, M173); bovine serum albumin (BSA) (Genetix, PG-2330); transfer buffer (Genetix, GX-9411AR), trizol reagent (Sigma-T9424), cDNA synthesis kit (Genetix-K1612). Caspase 3 (SC-4263) and caspase 8 (SC-4267) assay kits were procured from Santacruz Biotechnology Inc., California, Delaware. All others chemicals were of molecular biology grade and purchased from Genetix Biotech Asia Pvt. Ltd, New Delhi.

### Cell line and culture condition

The ER+MCF-7cells were cultured and routinely maintained in RPMI 1640 medium supplemented with 10% heat inactivated FBS, penicillin (100 units/ml), streptomycin (100μg/ml) and gentamycin (100μg/ml) in a humidified atmosphere at 37 °C containing 5% CO_2_ inside a CO_2_ incubator [63].

### Cytotoxicity study with MTT

Cytotoxicity was measured by using MTT assay. ER+MCF-7 cell lines (1x10^5^) were seeded in 96 well sterile plates and were treated with different concentrations of ALA (1μM, 5μM, 10μM, 15μM, 20μM, 25μM) for 24h, compared against control and standard (TMX) (27μM). Subsequently media form upper layer was removed and incubate with 20μl MTT (5 mg/ml) and 100μl of fresh media for 2h. Blue colored formazans were released from the cells by adding 100μl DMSO with gentle shaking at 37°C. After 30 min incubation the absorbance of the color solution was quantified by measuring at a wavelength of 570 nm by micro plate reader (Model 680 XR Bio-Rad laboratories lnc). IC_50_ value and ½ IC_50_ value of ALA against ER+MCF-7 cells were determined after 24 h [64].

### Detection of apoptosis through AO/EtBr staining

Characteristic apoptotic morphological changes were observed by inverted fluorescence microscopy by using AO/EtBr staining. ER+MCF-7 cells (1x10 ^6^) were treated with ½ IC 50 and IC50 dose of ALA and TMX for 18 h, and were observed under the inverted fluorescence microscope for morphological changes. The untreated control cells and ALA treated cells were harvested separately, washed with PBS and stained with AO (100μg/ml) and EtBr (100μg/ml) (1:1). The cells were immediately mounted on slides and visualized under an inverted fluorescence microscope (Nikon Leica, M165 FC) for the changes in morphological features of apoptosis [65].

### Identification of mitochondrial morphology through JC-1 dye

Alteration in the ΔΨm was analyzed by inverted fluorescence microscope using the mitochondrial membrane potential sensitive dye JC-1. JC-1 forms J-aggregate in control cells in intact mitochondrial membrane potential. Stock solution of 1 mg/ml JC-1 was prepared in 1% DMSO and stored at -20 °C shortly before use. After incubation for 24 h with ½ IC 50 and IC 50 dose of ALA, cells were washed three times with PBS and 200μl of JC-1(1 mg/ml) dye was added into each well. After incubation for 20 min, cells were washed three times with PBS and imaged with an inverted fluorescence microscope (Nikon Leica, M165 FC)[66].

### Cell cycle analysis using PI

The cells were treated with a fluorescent dye PI that quantitatively stains DNA. The amount of DNA was measured by the fluorescence intensity of the stained cells. For the investigation of cell cycle arrest stage, 1x10^6^ cells were treated with IC 50 and ½ IC 50 of ALA for 18 h. Cells were washed with PBS, fixed with methanol and kept at -20 °C for 3 min. Subsequently, cells were suspended in cold PBS and kept at 4 °C for 90 min. Cells were pelleted down, dissolved in PBS, treated with RNase for 30 min at 37 °C, stained with PI and kept in dark for 15 min. Cell cycle phase distribution of nuclear DNA was determined on florescence activated cell sorter (FACS), fluorescence detector equipped with 488 nm argon laser light source and 623 nm band pass filter (linear scale) using BD FACS software 1.2.0.87 (BD Influx cell sorter, USA)[63].

### Annexin-V FITC dot plot assay

Annexin-V FITC is a 35–36 kDa Ca^2+^dependent phospholipid-binding protein, which has high affinity for PS, and it binds to the exposed cell surface of apoptotic cell. The ER+MCF-7 cells (1X10^6^) were treated with individual IC50 and ½ IC 50 dose of ALA for 18 h. The cells were pellet down, centrifuged at 2000 rpm for 8 min at 4 °C and washed with annexin-V FITC binding buffer provided in apoptosis kit. After centrifugation at 2000 rpm at 4 °C, the cell pellets were dissolved in annexin-V FITC binding buffer containing annexin-V FITC and PI. The flow cytometric analysis was done after 15 min in dark at room temperature. All the data were acquired with a BD Influx cell sorter, USA. The reading were taken by using 488 nm excitation and band pass filters of 530/30 nm (for FITC detection) and 585/42 nm (for PI detection). For the alignment of X and Y mean values of annexin-V FITC or PI stained quadrant populations, live statistics were used. Data analysis was performed with BD FACS software 1.2.0.87 [67].

### Experimental protocol

Thirty two female albino wistar rats were randomized and divided into four groups of eight animals each. Animals were housed in standard condition (23°C, 12h light/dark cycle). The rats were obtained from central animal house facility of Babu Banarsi Das Northern India Institute of Technology, Lucknow and were housed in polypropylene cages. The animals were fed with standard pellet diet and water *ad libitum*. The experiment was performed according to the CPCSEA guidelines for laboratory animals and ethics, Department of animal welfare, Government of India (BBDNIIT/IAEC/021/2014). The animals were subjected to the treatment as Group I (control, normal saline, 1 ml/kg, p.o.), Group II (toxic, DMBA 8 mg/kg, i.v.), Group III (DMBA 8 mg/kg, i.v. + ALA 0.25ml/kg, p.o.) and Group IV (DMBA 8 mg/kg, i.v. + ALA 0.5ml/kg, p.o.). Toxicity was induced by single tail vein injection of DMBA on day 1. ALA was administered from 7^th^ to 110^th^ day at the dose specified above. The dose of ALA was selected from previous literature [68]. Tumors incidence and size were measured by the help of vernier caliper (Supplementary Table 2). Animals were sacrificed by cervical dislocation under light ether anesthesia on 112^th^day.

### Hemodynamic changes

The animals were anesthetized on 111^th^day of the study by using the combination of ketamine hydrochloride (50 mg/kg, i.m.) and diazepam (2.5 mg/kg, i.m.) and mounted on a wax tray. The platinum hook electrodes were placed on the skin of the dorsal and ventral thorax to record the ECG signal. The electrodes were connected to Bio-amplifier (ML-136) and channel power lab (ML-826) to convert analogue to digital signals (AD Instruments, Australia). The ECG signals were saved on the hard disk and analyzed offline using Lab Chart Pro-8 (AD Instruments, Australia)[69].

HRV analysis was conducted on multiple segments of continuous ECG signals. Firstly, all the raw signals were inspected manually to ensure that all the R-waves were detected correctly. Subsequently, HR was calculated by plotting the number of R waves per unit time. Following the same, time and frequency domain parameters of HRV were also calculated using Lab Chart Pro-8 (AD Instruments, Australia)[70].

### Morphological evaluation

#### Carmine staining of whole mounts mammary gland

The mammary glands obtained from female albino wistar rats were stretched onto a slide and kept in a fixative solution (60:30:10 ratio of ethanol: chloroform: acetic acid), stained with a carmine solution for 2 days, washed with 90%, 70%, 35% and 15% ethyl alcohol for 1 h respectively and lastly rinsed with distilled water for three times at 5 min interval. The tissue sample was dehydrated in ascending grades of alcohol and dipped in xylene at least for two days. Carmine alum stain was prepared with 1 gm carmine and 2.5 gm aluminum potassium sulfate in 450 ml distilled water and boiled for 20 min. The final volume was adjusted to 500 ml with distilled water. Whole mounts were examined under the 4X microscope and evaluated for ABs [24, 71].

#### Histopathology of mammary gland tissue

A small piece of mammary gland tissue was fixed in 10% solution of formaldehyde and embedded in the wax. 5μm sections were prepared using microtome followed by staining with H&E. The sections were visualized and photographed at 40X using digital biological microscope (N120, BR-Biochem Life Sciences, New Delhi, India)[72].

#### SEM of mammary gland tissue

The HCl-collagenase and enzymatic digestion methods were used for the purpose of SEM. Small sections of mammary gland tissues treated with HBSS containing 100μg/ml collagenase (type 4) and 2.5 TRU (turbidity reduction unit)/ml of hyaluronidase for 30 min at 37°C. After digestion, the tissue was rinsed in the HBSS and then fixed in 4% glutaraldehyde in 0.1 M cacodylate/HCl buffer (pH 7.2) at room temperature for 3 h. Post fixation was done with 1% osmium tetraoxide. Tissue sample was subsequently placed in 8 N HCl for 30 min at 60°C. After HCI digestion, the tissue was rinsed three times with distilled water to remove the acid. The sample was dried with 70%, 80%, 90% and 100% acetone. All the specimens were dehydrated, dried by the critical point method and examined under SEM(X1000)(JEOL JSM-6490LV)[25].

### Antioxidant markers

The mammary gland tissue homogenates (10% w/v) were prepared in 0.15 M KCl and centrifuged at 10,000 rpm. The supernatants were evaluated for oxidative stress markers, including TBARs, SOD, catalase, GSH and PC using the methods established in our laboratory [29, 73].

### ^1^H-NMR based serum metabolomics

#### Sample preparation for NMR spectroscopy

The stored serum samples were thawed at room temperature, 250 μl of serum was taken and mixed with 250 μl of sodium-phosphate buffer of strength 20 mM, pH 7.4 with 0.9% saline prepared in D_2_O [74](as a co-solvent and to provide a deuterium field/frequency lock). To remove any precipitates or cellular debris, the samples were further centrifuged at 10,000 rpm for 5 min at room temperature. The clear supernatant fluid of 400μl was finally used in 5 mm NMR tubes (Wilmad Glass, USA) for data acquisition with a co-axial insert containing the 0.1mM concentration of TSP (used here as external standard reference to aid spectral calibration). D_2_O and sodium salt of trimethylsilylpropionic acid-d4 (TSP) used for NMR experiments were purchased from Sigma-Aldrich (Rhode Island, USA).

#### NMR data acquisition

The ^1^H NMR spectra of all the samples were acquired on 800 MHz NMR spectrometer (BrukerAvance-III) equipped with the cryoprobe at 300 Kelvin (K). On each serum sample, the 1D ^1^H transverse relaxation-edited CPMG (Carr–Purcell–Meiboom–Gill) NMR spectra were recorded using the standard Bruker’s pulse program library sequence (cpmgpr1d) with pre-saturation of the water peak through irradiating it continuously during the recycle delay (RD) of 5 sec. Each spectrum consisted of the accumulation of 128 scans and lasted for approximately 15 min. To remove broad signals from proteins and fats, a total spin–spin relaxation time of 60 ms (n=300 and 2τ=200μs) was applied. Each FID (free induction decay) was zero filled and Fourier-transformed to 64 K data points following manual phase and baseline-correction. A line broadening factor of 0.3 Hz and a sine–bell apodization function was applied to FIDs before Fourier Transformation (FT). The raw NMR data were processed using Bruker software Topspin-v2.1 (BrukerBioSpin GmbH, Silberstreifen 4 76287 Rheinstetten, Germany).

#### Assignment of the ^1^H NMR spectra

The metabolite resonances in the 1D ^1^H CPMG NMR spectra were assigned using the Chenomx NMR suite (Chenomx Inc., Edmonton, AB, Canada). The remaining peaks in the CPMG ^1^H NMR spectra were assigned as far as possible, by comparing them with the chemical shifts available using previously reported NMR assignments of metabolites [75, 76], data obtained from BMRB database (Biological Magnetic Resonance Data Bank [77] and HMDB (The Human Metabolome Database [78].

#### Multivariate statistical analysis

The ^1^H NMR spectra of all the serum samples were manually phase adjusted and baseline corrected after referencing to the alanine resonance at δ(1.46) ppm. The CPMG δ 9.5-0.7 ppm spectra were binned and automatically integrated using AMIX package (Version 3.8.7, Bruker, BioSpin), to reduce the complexity to the NMR data and facilitate pattern recognition. The region distorted due to water suppression δ (5.5-4.5) ppm, were excluded from the CPMG data set. Finally, the selected regions were reduced to spectral bins of δ 0.01 ppm. The resultant CPMG, data sets were eventually used for univariate and multivariate analysis in statistical analysis module of MetaboAnalyst, an open access web-based tool for metabolomics studies [79, 80].

Using standard procedures for multivariate statistical analysis in MetaboAnalyst, PCA, PLS-DA, and OPLS-DA were performed on all the groups to get an overview of the grouping trends and to separate the effective treatment dose. The PLS-DA model was further used for pairwise analysis to identify the metabolites responsible for discrimination based on their higher values of variable importance on projection scores (i.e. VIPs > 1). Furthermore, unpaired t-test was applied to assess the significance of change in the metabolic profile and p-value < 0.05 was used as the criterion for statistical significance. Metabolites meeting the above said criteria were considered to be significant. A 10-fold cross-validation algorithm using the top 5 latent variables, was used –which helped to evaluate 100% classification accuracy, along with the goodness-of fit parameter (R^2^) and the goodness of prediction parameter (Q^2^) values to assess the quality (or predictability) of the models, respectively.

### Assay for caspase 3 and caspase 8

Caspase 3 and caspase 8 fluorometric assays were performed using the methods elaborated in the literature provided with the kits. The assay was carried out in amber colored 96-well plate. Equal volumes of serum sample from both control and experimental animals were diluted with reaction buffer. Dithiothriol (DTT) was added to a final concentration of 10mM. To the reactant mixture 5μl of IETD-AFC/DEVD-AFC substrate was added and incubated for 1 h at 37°C. Free AFC levels formed were measured in a plate reader with a 400 nm excitation and a 505 nm emission. The results were expressed as fluorescence units/mg of protein [81].

### Western blotting

Total protein lysates were obtained by lysing the mammary gland tissue in RIPA lysis buffer. The protein content was quantified using the Bradford reagent [82]. According to the principles of Laemmli with slight modifications, proteins were resolved on 12.5% SDS-PAGE gel and transferred to PVDF membrane (IPVH 00010 Millipore, Bedford, MA USA. Subsequently, membrane was blocked with 3% BSA and 3% not fat milk in TBST for 3 h and incubated overnight with primary antibody against, Bcl-xl (MA-5-15142), Bcl-2 (SC-7382), BAX (SC-23959), BAD (SC-8044) VDAC (SC-390996), cytochrome c (SC-13561), Apaf-1 (SC-65891), procaspase 9 (SC-73548), NFκBp65 (MA5-1616), UCHL-1 (MA1-83428), PHD2 (SC-67030), HIF-1α (SC-13515), FASN (SC-55580), SREBP-1c (SC-13551)at 4°C. β-actin (MA5-15739-HRP) was used as a standard reference. The membrane was washed with TBST thrice and incubated with the corresponding anti-rabbit (SC-2030), anti-goat (SC-2020), anti-mouse (31430, Pierce Thermo Scientific, USA) HRP conjugated secondary antibody (1:5000 dilutions) at room temperature for 3 h. After single TBST wash membranes were developed using an enhanced chemiluminescence substrate (Western Bright ECL HRP substrate, Advansta, Melanopark, California, US) in gel dock system. The quantification of protein was done through densitometry digital analysis of protein bands using Image J software [83, 84].

### qRT-PCR

Primers for real time were designed online using primer quest tool from the IDT DNA technologies website (www.idtdna.com). The amplicon size was kept between 100 to 200 base pairs, GC% was kept above 50% and melting temperature was kept between 58°C to 62°C. The specific sequences of the forward and reverse primers are specified in Supplementary Table 3.

Total RNA was extracted from mammary gland tissue using trizol reagent according to the manufacturer's instructions. Briefly, tissues were washed off treatment plates using 0.1% DEPC water. The tissues were crushed in 250μl trizol reagent using micro pestles. Another 750μl of trizol reagent was added to make the final volume to 1ml, followed by addition of 200μl of chloroform and mixing for 2 to 5 min on a vortex mixer. The suspension was then centrifuged at 14000rpm, 4°C for 15 min and upper aqueous phase was gently pipette out in the fresh vials. RNA was precipitated by addition of 500μl chilled isopropanol. The vials were kept at room temperature for 10 min and were centrifuged at 14000rpm, 4°C for 10 min and RNA pellet so obtained was washed twice with 75% ethanol (chilled) at 7500rpm, 4°C for 5 min. The RNA pellet was finally dissolved in 15μl of 0.1% DEPC water. To quantify RNA absorbance was read using nano drop (Qua Well Q5000).

cDNA synthesis was done from 1μg of total mammary gland RNA in a 96 well thermal cycler (BioRad, C1000) with steps including, incubation at 25°C for 10 min, 37°C for 120 min, 85°C for 5 min and 4°C forever RNA using high capacity cDNA synthesis kit. cDNA sample were quantified using nanodrop and were stored at -80°C until use. 125 ng of cDNA was used as template for each reaction of qRT-PCR with β-actin as housekeeping control using light cycler 480 machine (Roche Diagnostics, Germany). For each primer pair, a melting curve analysis was performed according to instrument. The program in brief was an initial incubation of 50°C for 2 min hold (UDG incubation) and 95°C for 10 min followed by 40 cycles at 95°C for 15 s (denaturation), 58°C for 30 s (annealing) and final extension at 72°C for 20 s. Differential expression was calculated by 2-∆∆CT method. β-actin was used as internal control and used to normalize ratios between samples [85, 86].

### Statistical analysis

All data were presented as mean ± SD and analyzed by one-way ANOVA followed by Bonferroni test and for the possible significance identification between the various groups. *p<0.05, **p<0.01, ***p<0.001 were considered as statistically significant. Statistical analysis was performed using Graph Pad Prism software (5.02).

## CONCLUSION

To conclude, authors would like to submit that ALA ameliorates the morphological, biochemical and associated biological effects of DMBA. As hypothesized, ALA persuaded the mitochondrial mediated death pathway to impede the hypoxic microenvironment and curtail de novo fatty acid synthesis. Our results suggest the possible therapeutic potential of ALA against mammary gland carcinoma without any untoward effect. The study also validates the need of clinical evaluation of ALA for its future use.

## SUPPLEMENTARY MATERIALS FIGURES AND TABLES



## ABBREVIATIONS

ALA: α-Linolenic acid
AA: arachidonic acid
AO: acridinie orange
AB: alveolar bud
BSA: bovine serum albumin
BMRB: biological magnetic resonance data bank
CPMG: carr–purcell–meiboom–gill
CEC: cuboidal epithelial cell
DHA: docosahexaenoic acid
DMSO: dimethyl sulphoxide
DMBA-7: 12-dimethylbenz(a)anthracene
DTT: dithiothriol
DCT: dense connective tissue
EPA: eiconapentanoic acid
EBSS: eagle balanced salt solution
EtBr: ethidium bromide
ECG: electrocardiogram
FBS: fetal bovine serum
FACS: florescence activated cell sorter
FID: free induction decay
FT: fourier transformation
FASN: fatty acid synthase
GSH: glutathione
GPC: glycerophosphocholine
HBSS: hank’s balanced salt solution
H&E: hematoxylin & eosin
HRV: heart rate variability
HR: heart rate
HMBD: the human metabolome database
HF: high frequency
HIF: 1α- hypoxia inducible factor-1α
LF: low frequency
LCT: loose connective tissue
LDL: low density lipoprotein
MTT: 3-(4, 5-Dimethyl-2-thiazolyl)-2, 5-diphenyl-2H-tetrazolium bromide
MPTP: mitochondria permeability transition pore
MEC: mypepithelial cell
NAG: N-acetyl glycoprotein
OAG: O-acetyl glycoprotein
PUFA: polyunsaturated fatty acid
PI: propidium iodide
PS: phosphatidylserine
PC: phosphocholine
PCA: principal component analysis
PLS-DA: partial least squares discriminant analysis
PHD2: prolyl hydroxylase-2
RD: recycle delay
ROS: reactive oxygen species
SEM: scanning electron microscope
SOD: superoxide dismutase
TMX: tamoxifen citrate
TRU: turbidity reduction unit
TBARs: thiobarbituric acid reactive substances
TSP: trimethylsilylpropionic acid-d4
VLF: very low frequency
VLDL: very low density lipoprotein
