# Supplementary material for: Alpha-linolenic acid stabilizes HIF-1 α and downregulates FASN to promote mitochondrial apoptosis for mammary gland chemoprevention
Source: Oncotarget. 2017 Jul 25;8(41):70049–71. doi: 10.18632/oncotarget.19551 (PMC5642536; doi:10.18632/oncotarget.19551)
Supplement: Supplementary file 1 [file oncotarget-08-70049-s001.pdf]

Alpha-linolenic acid stabilizes HIF-1 α and downregulates FASN to promote mitochondrial apoptosis for mammary gland chemoprevention

SUPPLEMENTARY MATERIALS

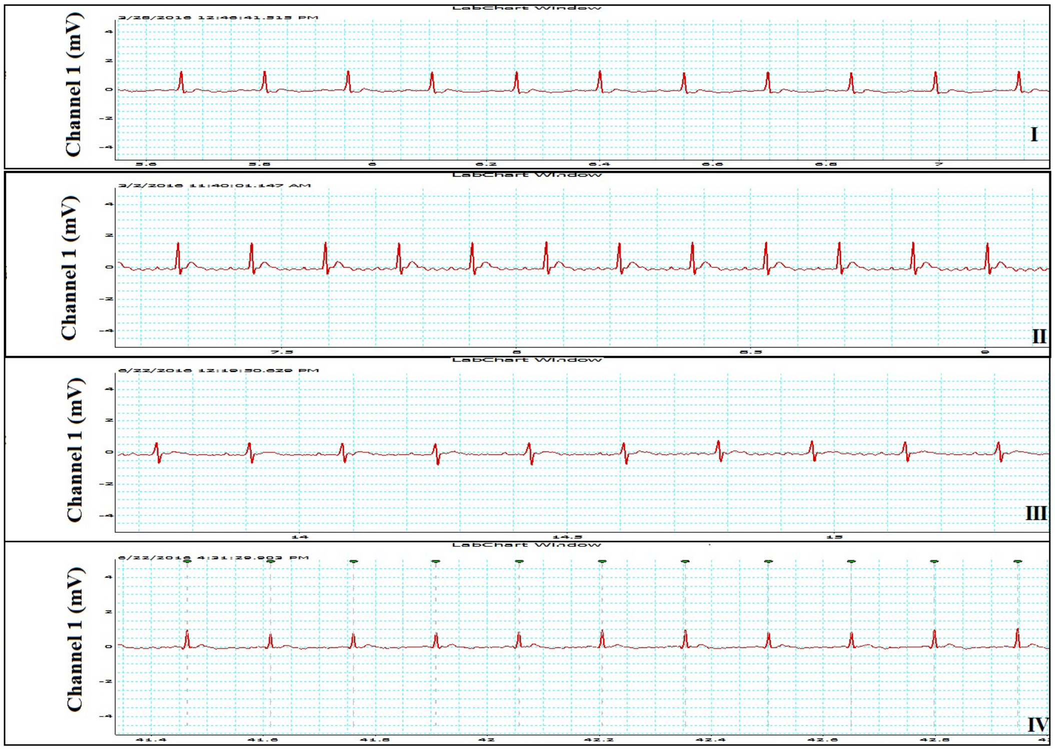

Supplementary Figure 1: ECG recording. Group I: normal control (0.9% normal saline, p.o.), Group II: toxic control (8 mg/kg DMBA, i.v.), Group III: (8 mg/kg DMBA, i.v. + 0.25 ml/kg ALA, p.o.), Group IV: (8 mg/kg DMBA, i.v. + 0.5 ml/kg ALA, p.o.).

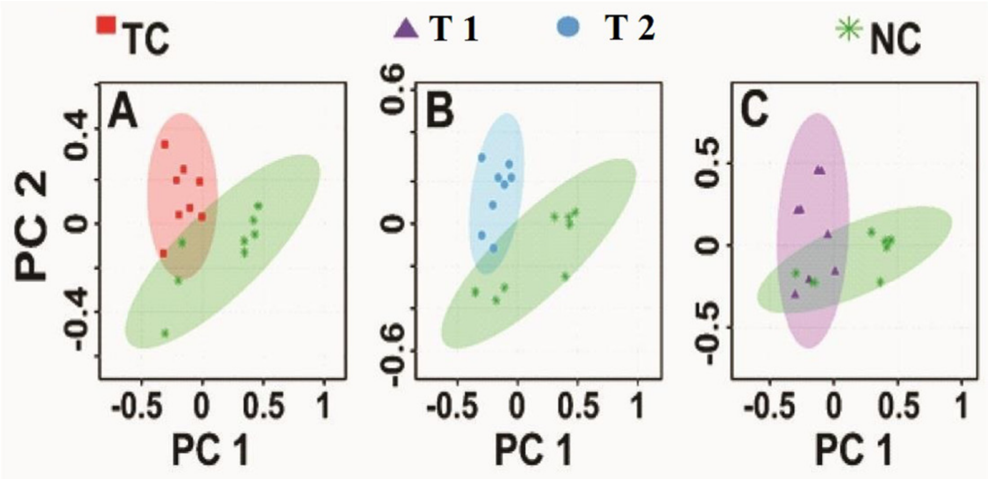

Supplementary Figure 2: The 2D PCA score plots for pairwise analysis. In **(A)**, normal control (NC) and DMBA treated toxic control (TC) group in **(B)**, between NC and DMBA+0.25ml/kg-ALA, and in **(C)**between NC vs DMBA+ALA-0.5ml/kg.

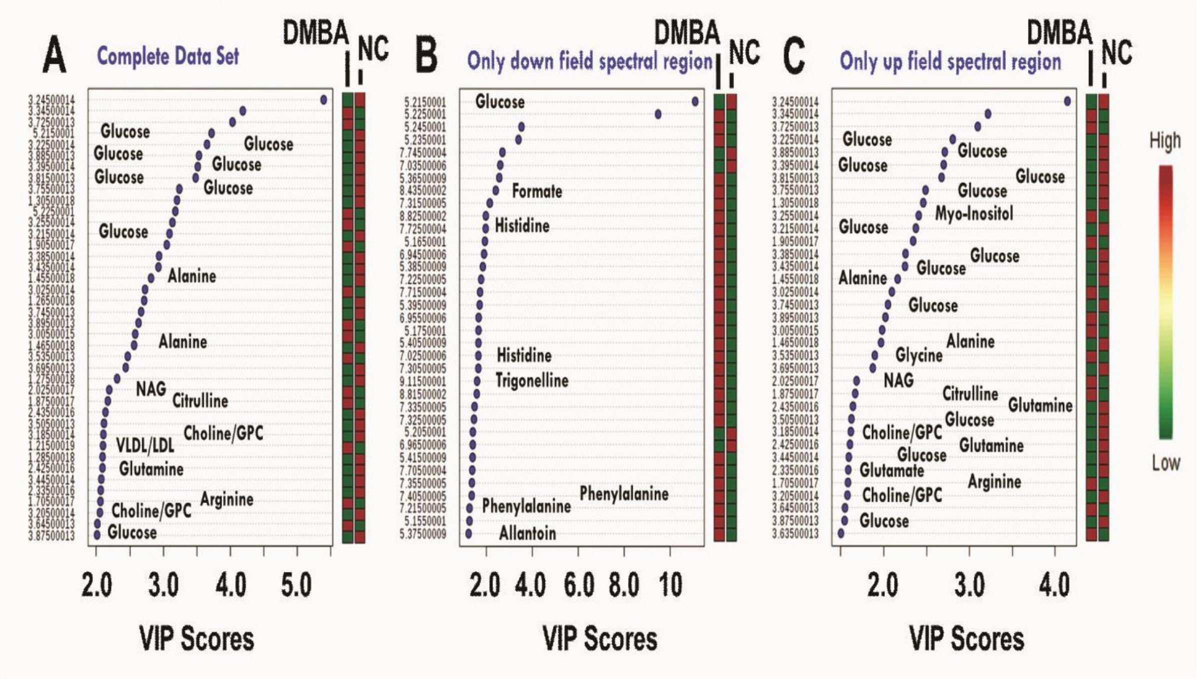

Supplementary Figure 3: The potential biomarker metabolite entities identified from PLS-DA analysis and are listed in decreasing order of VIP score to highlight their discriminatory potential. In **(A)**, the complete NMR data matrix was used to PLS-DA modeling and resulted VIP scores for top 35 metabolite entities are shown. In **(B)**, the down-field spectral region from 5.4 to 9.5 ppm was used for PLS-DA modeling and revealed the discriminatory importance of aromatic amino acids like Histidine, Tyrosine and phenylalanine. In **(C)**, the up-field spectral region from 0.9 to 4.5 ppm was used for PLS-DA modeling and revealed the discriminatory importance of other serum metabolites mainly amino acids and metabolites of tricarboxylic acid cycle.

Supplementary Table 1: Effect of ALA on electrocardiographic changes DMBA induced mammary gland carcinoma

ECG parameters	Control (0.9% normal saline, p.o)	Toxic control (DMBA 8 mg/kg, i.v.)	DMBA +ALA (8 mg/kg i.v. +0.25 ml/kg, p.o.)	DMA + ALA (8 mg/kg i.v. +0.5 ml/kg, p.o.)
**RR Interval (s)**	0.17±0.01	0.18±0.01	0.20±0.009**	0.17±0.01
**Heart Rate (BPM)**	331.3±0.17**	353.2±0.42	306.6±0.09***	297.8±0.79***
**PR Interval (s)**	0.04±0.002	0.04±0.002	0.04±0.003	0.05±0.004***
**P Duration (s)**	0.01±0.004	0.01±0.001	0.01±0.001	0.01±0.002
**QRS Interval (s)**	0.01±0.004	0.01±0.004	0.01±0.003	0.02±0.006***
**QT Interval (s)**	0.04±0.01	0.05±0.08	0.06±0.09	0.05±0.01
**QTc (s)**	0.1±0.02	0.1±0.01	0.1±0.02	0.1±0.02
**JT Interval (s)**	0.02±0.01	0.04±0.01	0.04±0.1	0.03±0.01
**T peak Tend Interval (s)**	0.01±0.007	0.02±0.009	0.02±0.007	0.01±0.005
**P Amplitude (mV)**	0.05±0.01	0.09±0.03	0.02±0.2	0.05±0.07
**Q Amplitude (mV)**	0.03±0.03	0.02±0.01	0.007±0.03	0.005±0.07
**R Amplitude (mV)**	1.4±0.48**	2.0±0.2	0.7±0.2 ***	0.9±0.4***
**S Amplitude (mV)**	-0.2±0.1	-0.1±0.06	-0.1±0.05* -	0.09±0.2
**ST Segment (mV)**	-0.08±0.1	0.07±0.06	0.04±0.05 -	0.04±0.08
**T Amplitude (mV)**	0.35±0.06	0.34±0.1	0.15±0.1**	0.12±0.1***

(Values are presented as Mean ± SD). Each group contains eight animals. Comparisons were made on the basis of the one-way ANOVA followed by Bonferroni multiple test. All groups were compared to the toxic control group (*p<0.05, **p<0.01, ***p<0.001).

Supplementary Table 2: Effect of ALA on DMBA induced mammary gland carcinoma in female albino wistar rats

Treatment groups	No. of rats with tumors/total rats	Tumor incidence (%)	Total tumor burden (cm^3)
Control; 0.9% normal saline, p.o.	0/8	-	-
Toxic control; DMBA 8 mg/kg, i.v.	6/8	75	380.55
DMBA 8 mg/kg, i.v.+ ALA 0.25 ml/kg, p.o.	3/8	37.5	180.75
DMBA 8 mg/kg, i.v. + ALA 0.5 ml/kg, p.o.	1/8	12.5	110.05

Rats from normal (Group I, n =8) did not show any visible mammary tumor. Group II have high no. of visible tumors which were subsided by ALA treatment.

Supplementary Table 3: Sequence of forward and reverse primers used for quantitative RT-PCR

Primer	Sequence
Bcl2 F	GTGGATGACTGAGTACCTGAAC
Bcl2 R	GAGACAGCCAGGAGAAATCAA
Bcl-xl F	CCCTCGTATCTGGAAGCCAC
Bcl-xl R	CAGCGGAGACCTCGTTTTCT
BAX F	TGCTACAGGGTTTCATCCAG
BAX	RGACACTCGCTCAGCTTCTT
BAD F	CTCCGAAGAATGAGCGATGAA
BAD R	ATCCCACCAGGACTGGATAA
VDAC F	GGAGTTTGGTGGCTCCATTTA
VDAC R	GACCTGATACTTGGCTGCTATTC
Cytochrome-c F	TCCATTTCCCTTCCTTGGGC
Cytochrome-c R	ATCGGGGCTGTCCAACAAAA
Apaf-1F	GAACATAGACTCCCGGGTAAAG
Apaf-1R	CTTGTCTCCCAGACCCTTATTG
Procaspase9F	GGCTCTCTGGCTTCATTCTT
Procaspase9R	GGGTCCAGCTTCACTACTTTC
PHD2 F	ACGCAGTTCATACCCAGTTAG
PHD2 R	CCTGTCCACTCTCAGCTTTAC
HIF1-α F	GATGGGTTATGAGCCAGAAGAA
HIF1-α R	CTGTGGTGACTTGTCCTTTAGT
FASN F	GGCGAGTCTATGCCACTATTC
FASN R	GCTGATACAGAGAACGGATGAG
SREBP-1cF	TCCGAGTTCCAGGTAGGGTT
SREBP-1cR	CTTGGCGCACACCAAATACC
UCHL-1 F	CGCCTCTGCCCTGAGTTATT
UCHL-1 R	CCGTCTGGGTCAATCCTCTG
NFκBp65 F	GGGCTACGAAGTCAAACCCA
NKκBp65	RTTCTCCTCAATCCGGTGACG
β-actin	FTGCAGGATCGTGAGGAACAC
β-actin R	AGCGTGATTGTAACGCCTGA
